# Utilization of innovative medical technologies in German inpatient care: does evidence matter?

**DOI:** 10.1186/s12961-023-01047-w

**Published:** 2023-10-02

**Authors:** Helene Eckhardt, Susanne Felgner, Marie Dreger, Sabine Fuchs, Hanna Ermann, Hendrikje Rödiger, Tanja Rombey, Reinhard Busse, Cornelia Henschke, Dimitra Panteli

**Affiliations:** 1 Department of Health Care Management, Technische Universität Berlin, Straße Des 17. Juni 135, 10623 Berlin, Germany; 2 Berlin Centre for Health Economics Research (BerlinHECOR), Technische Universität Berlin, Straße Des 17. Juni 135, 10623 Berlin, Germany

**Keywords:** Medical devices, Evidence-based medicine, Value based health care, Evidence gaps, Implementation science, Diffusion of innovation, Health technology assessment, Financial incentives, Disinvestment, Hospital, Germany

## Abstract

**Background:**

The reimbursement of new technologies in inpatient care is not always linked to a requirement for evidence-based evaluation of patient benefit. In Germany, every new technology approved for market was until recently eligible for reimbursement in inpatient care unless explicitly excluded. The aim of this work was (1) to investigate the type of evidence that was available at the time of introduction of 25 innovative technologies and how this evidence evolved over time, and (2) to explore the relationship between clinical evidence and utilization for these technologies in German inpatient care.

**Methods:**

This study combined different methods. A systematic search for evidence published between 2003 and 2017 was conducted in four bibliographic databases, clinical trial registries, resources for clinical guidelines, and health technology assessment—databases. Information was also collected on funding mechanisms and safety notices. Utilization was measured by hospital procedures captured in claims data. The body of evidence, funding and safety notices per technology were analyzed descriptively. The relationship between utilization and evidence was explored empirically using a multilevel regression analysis.

**Results:**

The number of included publications per technology ranges from two to 498. For all technologies, non-comparative studies form the bulk of the evidence. The number of randomized controlled clinical trials per technology ranges from zero to 19. Some technologies were utilized for several years without an adequate evidence base. A relationship between evidence and utilization could be shown for several but not all technologies.

**Conclusions:**

This study reveals a mixed picture regarding the evidence available for new technologies, and the relationship between the development of evidence and the use of technologies over time. Although the influence of funding and safety notices requires further investigation, these results re-emphasize the need for strengthening market approval standards and HTA pathways as well as approaches such as coverage with evidence development.

**Supplementary Information:**

The online version contains supplementary material available at 10.1186/s12961-023-01047-w.

## Background

Health-related technological progress plays an important role in the improvement of health outcomes. Yet new technologies may also bear risks to patients and users [1, 2]. Ever since the Contergan (Thalidomide) scandal in the 1950s [3], market access for pharmaceuticals is highly regulated and generally requires extensive clinical evaluation. The approval process for new medical devices in the European context is decentralized and entails verifying the conformity of a device with the European Union (EU) regulatory framework, primarily regarding its intended use and safety. The necessity of clinical investigation to determine the efficacy and safety of new implantable or high-risk medical devices for market approval was introduced in 2007 (Directive 2007/47/EC [4]). Following repeated reports of patient harm, the Medical Device Regulation (MDR) (Regulation (EU) 2017/745 [5]), which aimed to reform the EU regulatory framework on medical devices (Directive 93/42/EEC [6] and Directive 90/385/EEC [7]), introduced the term “clinical benefit” as a criterion for the approval of medical devices for the first time [8]. In contrast, the premarket approval process by the Food and Drug Administration (FDA) in the United States (US) has required evidence from clinical trials to determine effectiveness and safety of innovative high-risk medical devices since the early 1990s [9–11].

Once they have been approved for market, the pathway to reimbursement of medical technologies in European health systems generally varies [12–14]. While health technology assessment (HTA) to determine comparative (cost-)effectiveness of new technologies is often a prerequisite for pharmaceuticals in the outpatient sector, this is not always the case for other technologies or the inpatient setting [13]. However, innovative, high-risk and high-cost medical devices are more frequently the subject of HTA linked to reimbursement [15]. This is not always the case in Germany, where traditionally every new technology for diagnosis or treatment (*Neue Untersuchungs- und Behandlungsmethode, NUB)* has been reimbursable in inpatient care unless explicitly excluded by the Federal Joint Committee (FJC; § 137c German Social Code Book V [*SGB V*]). When the costs for such NUBs cannot yet be sufficiently accounted for by an existing diagnosis-related group (DRG), hospitals can apply to the German Institute for the Hospital Remuneration System (InEK) for the permission to negotiate one-year, hospital-individual extra-budgetary payments (also referred to as “innovation payments”) with health insurers [16, 17]. Once these new technologies have been included in the German Procedures Classification [16], their adequate cost-based funding is achieved either through supplementary payments (fixed or negotiable) considered in the annual budget negotiations between hospitals and health insurers, the split of an existing DRG or the creation of a new one. *Fixed supplementary payments* entail a uniform national price per case published in the appendix of the DRG catalogue; when a uniform national price cannot be determined, hospitals can still obtain individual (confidential) *negotiable supplementary payments* with a minimum amount of € 600 per case [17].

Against the background of a lacking requirement for HTA prior to reimbursement and the described financial incentives, it is important for quality of care and value for money to enquire whether the implementation of innovative technologies in German inpatient care is guided by clinical evidence. Previous research on the influence of evidence or funding mechanisms on implementation and diffusion has largely focused on individual technologies [17–19] or on coverage decisions [20].

The overall aim of this work was twofold: Firstly, we aimed to investigate the type of evidence that was available at the time of introduction of selected innovative technologies in German inpatient care and how this evidence evolved over time. Secondly, we sought to explore the link between the available scientific evidence, such as clinical trials, systematic reviews, or health technology assessment reports, and the diffusion of these technologies.

This exploratory analysis consisted of three steps: (i) the systematic identification of scientific evidence regarding the safety and efficacy/effectiveness for each technology, (ii) the description of the available body of evidence for each technology at the time of introduction and its development over time; and (iii) the investigation of the statistical relationship between the identified evidence and the utilization of technologies by hospitals. Additionally, the potential impact of clinical guidelines, funding mechanisms and safety notices (warnings and recalls) on the utilization of these technologies was investigated based on step (ii), above, to facilitate a more contextualized interpretation.

## Methods

### Selected technologies and data on utilization

The pool of eligible technologies comprised those for which requested permissions for hospital-individual extra-budgetary payments were granted between 2005 and 2012. This time window ensured the availability of data for at least five years preceding the start of this research in 2018 (i.e., observation period for this study: 2005–2017). Utilization curves for each technology were plotted on the basis of data identified via the respective procedure codes from the DRG statistics dataset. This dataset is made available by the Research Data Centre (RDC) of the Federal Statistical Office and Statistical Offices of the Federal States [21] upon formal request and prior agreement. For the purpose of this analysis, data on the number of inpatient procedures per technology, per hospital and per year were provided by the funder. A technology was included in the analysis if it met the following criteria: at least ten hospitals requested and obtained permission for hospital-individual extra-budgetary payments for a minimum of one year between 2005 and 2012, procedure numbers and the number of hospitals using the technology were available for at least four years, and there were at least 100 procedures performed for a minimum of one year. Based on these criteria, 59 technologies were shortlisted and a quantitative cluster analysis was applied to narrow down different types of utilization profiles. Finally, 25 technologies flowed into the analysis (this process is described in detail elsewhere [22]). This approach aimed to ensure that the final sample included both technologies with large utilization volumes and those with inconsistencies in utilization patterns. Procedure codes for the 25 technologies and their descriptions are provided in the Appendix 1 of the Additional file 1.

### Search for information on funding, recalls and safety warnings

Information sources on funding, recalls and safety warnings per technology are listed in Additional file 1, Appendix 2.

### Search for clinical evidence

A systematic literature search was carried out in 2019 in PubMed, Medline (via OVID), Embase (via OVID) and the Cochrane Library. The development of search strategies for each included technology was based on the PICO framework (population, intervention, comparison, outcomes). To define search terms, appraisals by the Medical Service of the National Association of Health Insurance Funds and the Procedure Classification were consulted, focusing on the intervention (technology-specific terms, product, and manufacturer names) and the indication (technology specific search strategies are available upon request). Supplementary searches were performed in the reference lists of included systematic reviews, clinical trial registries, HTA databases and clinical guideline databases (details in Additional file 1, Appendix 2). EndNote X9 files were created per technology and used for duplicates removal [23], after search results were imported, and for documentation of the screening process.

### Selection of evidence

General inclusion and exclusion criteria based on the PICO framework were developed to select relevant evidence for each technology (full details in Additional file 1, Appendix 3, table S3.1). Published and unpublished studies belonging to levels of evidence (LoE) 1–4 according to the definition of the FJC (2. Chapter, §11 [3], procedure rules of FJC) [24] were included, while studies belonging to LoE 5 were documented, but not analyzed further:

- 1a Systematic reviews of randomized controlled trials
- 1b Randomized controlled trials (RCT)
- 2a Systematic reviews of non-randomized controlled trials
- 2b Prospective non- randomized controlled trials (N-RCT)
- 3 Retrospective controlled trials
- 4 Case-series and other single-arm trials
- 5 Case reports, etc.

Publications starting two years before the first documentation of hospital procedures (earliest 2003) and up to 2017 were eligible for inclusion.

Evidence was selected in line with the rapid review methods of the Cochrane Collaboration [25]. After duplicate removal, a random sample of 10% of citations (min. 100) per technology was drawn via RStudio (Version 1.4.1717). Two researchers independently screened the sample and selected relevant citations. In case of discrepancies, the inclusion and exclusion criteria were discussed and adjusted, involving a third researcher if necessary. The remaining citations were screened by one person based on the adjusted criteria. Each review step was documented as recommended by the PRISMA statement [26].

The selection of evidence identified in trial registries, guideline and HTA-databases, and the process of data extraction and risk of bias (RoB) assessment are described in Additional file 1 Appendices 4, 5 and 6 respectively.

### Categorization of study results

**Fig. 1:**
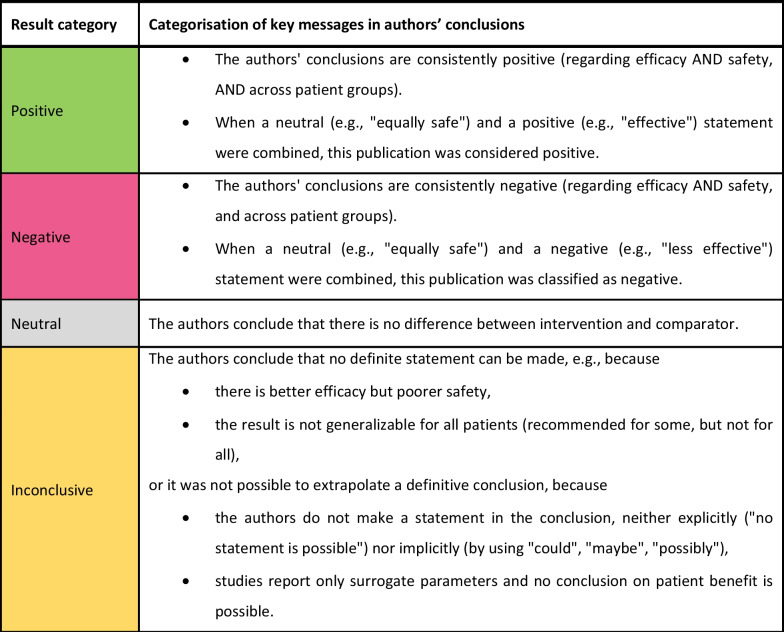
Categorization scheme of key messages in authors’ conclusions in identified publications. Note: Authors’ own elaboration, inspired by the GRADE approach [27, 28]

For each publication, authors’ conclusions were extracted from abstract and main text and categorized into “positive,” “negative,” “neutral,” or “inconclusive” according to the categorization scheme described in Fig. 1. If no conclusion section in the main text was available, the categorization was performed based on the summary of results from the discussion section and from the abstract.

### Synthesis and statistical analysis

To explore (a) the characteristics of the available body of evidence for each of the included technologies at the point of their introduction and over time and (b) the potential relationship between evidence and utilization patterns, both a descriptive and a statistical analysis were conducted.

*Descriptive analysis:* The identified body of evidence was analyzed descriptively. Each publication was considered as a separate data point. The recommendations from clinical guidelines, information on funding and safety notices were synthesized narratively for each technology and are discussed in combination with other findings.

*Empirical evaluation of the relationship between utilization and evidence results*: To evaluate whether utilization of a technology over time follows available clinical evidence, a new variable X, *“results of available body of evidence”* at year t and technology j, was aggregated. The variable incorporates all identified comparative analyses (LoE 1–3) of a technology published up to and including year t, weighted by category of study results (positive, negative, neutral) and LoE. The variable depicts the prevailing results of the body of evidence available starting two years before the beginning of utilization in Germany cumulated over all years of utilization until year t. Data for the outcome variable “utilization” is represented by the number of hospital procedures in year t and technology j.

Due to the clustered data structure and to account for technology-specific effects, a mixed effects model (multilevel model) for two levels of data was applied [29, 30]. It is described in more detail in Additional file 1, Appendix 7. The aim of the regression function is to estimate whether the development of utilization follows the direction of study results, but not to explain the whole variance; this would not be possible by including only one explanatory variable, but most other potentially influencing factors [31] are difficult to quantify. The regression analysis was performed using Rstudio (Version 1.4.1717) and the lme4 package.

## Results

### Results of evidence search

**Fig. 2:**
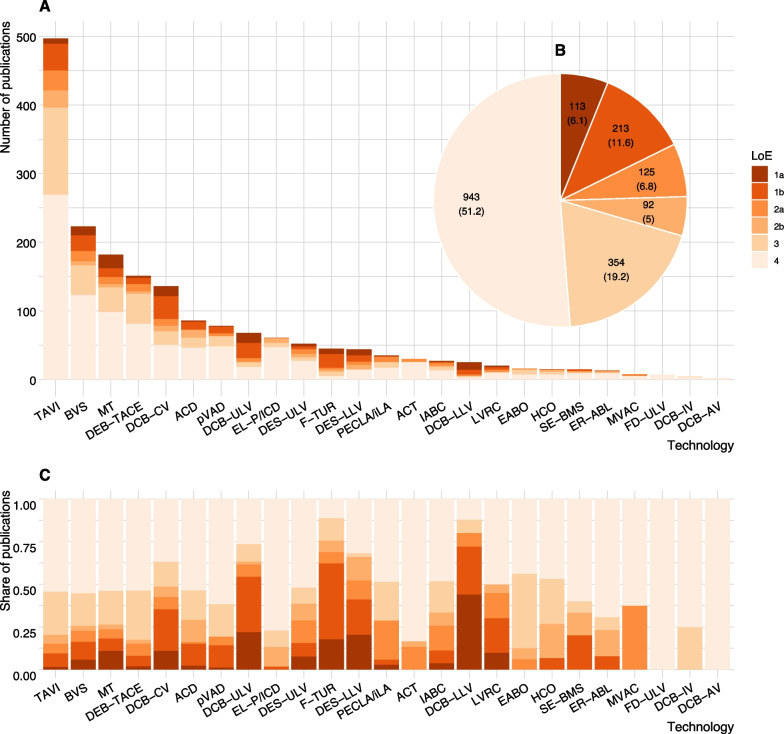
Number of publications (**A**) by level of evidence (LoE) and technology, (**B**) cumulated by LoE over all technologies [*n* (%)], and (**C**) shares of publications by LoE and technology. Source: Created by the authors; ACD—Anticoagulation with citrate during dialysis; ACT—Adjustable continence therapy; BVS—Bioresorbable Vascular Scaffold in coronary vessels; DCB-AV—Drug-coated balloon catheter in abdominal vessels; DCB-CV—Drug-coated balloon catheter in coronary vessels; DCB-IV—Drug-coated balloon catheter in intracranial vessels; DCB-LLV—Drug-coated balloon catheter in lower leg vessels; DCB-ULV—Drug-coated balloon catheter in upper leg vessels; DEB-TACE—Drug-eluting beads for trans-arterial chemoembolization; DES-LLV—Implantation of a drug-eluting stent in lower leg vessels; DES-ULV—Implantation of a drug-eluting stent in upper leg vessels; EABO—Endo-aortic balloon occlusion with extracorporeal circulation; EL-P/ ICD—Excimer laser extraction of pacemaker and defibrillator electrodes; ER-ABL—Cardiac event recorder after ablative measures for atrial fibrillation / atrial tachycardia; FD-ULV—Flow-diverter (Hemodynamically effective implant for endovascular treatment of peripheral aneurysms) in upper leg vessels; F-TUR—Fluorescence-assisted transurethral resection; HCO—Dialysis with high cut-off dialysis membrane; IABC—Bioactive coils for intracranial aneurysm therapy; LVRC—Lung volume reduction by insertion of coils; MT—Intracranial endovascular mechanical thrombectomy; MVAC—Mitral valve annuloplasty with clamp; PECLA/ iLA—Pumpless Extracorporeal Lung Assist/ Interventional Lung Assist; pVAD—Percutaneous ventricular assist device (Micro-axial blood pump); SE-BMS—Self-expanding bare metal stents in coronary vessels; TAVI—Transcatheter aortic valve implantation

All searches yielded citation numbers in the four-digit range. The number of publications included in the final analysis (LoE 1–4) ranges from two (drug-coated balloon catheter in abdominal vessels—DCB-AV) to 498 (transcatheter aortic valve implantation—TAVI) (Fig. 2). Additional file 1, Appendix 8, table 8.1 shows the results of the searches and screening by technology, bibliographic database, and step within the selection process.

Results of the searches for grey literature (clinical guidelines, HTA reports, trial registry entries and safety notifications) are presented in Additional file 1, Appendix 8, table 8.2. Across technologies and years, 40 HTA reports, and 40 clinical guidelines were identified. The number of HTA reports per technology varies between zero and 12. Clinical guideline recommendations were identified for 19 technologies; the number of guidelines (and their updates) varies between zero and 13 (fluorescence-assisted transurethral resection—F-TUR clinical guideline with 12 annual updates between 2006 and 2017). Further results on clinical guidelines are presented in Additional file 1, Appendix 12.

At least one safety notification was identified for 12 technologies in Germany and for two further technologies internationally. The number of safety notifications in Germany ranges from one (pumpless extracorporeal lung assist/ interventional lung assist—PECLA/iLA) to 12 (TAVI). Internationally, TAVI and bioresorbable vascular scaffold in coronary vessels (BVS) are the technologies with the most notifications (74 notifications for TAVI and 48 for BVS). At least one recall was identified for seven technologies in Germany and for six additional technologies internationally.

### Characteristics of the body of evidence

The composition of the body of evidence per technology is shown in Fig. 2A. For almost all technologies the bulk of the evidence consists of case series and other non-comparative studies; these designs make up more than half of all identified publications across technologies (943/1840) (Fig. 2B and C). Only 213/1840 (12%) of included publications report results from 130 individual RCTs. No RCT was identified for six out of 25 technologies. For the remaining 19 technologies, the number of RCTs with at least one publication per technology varies from one (e.g., PECLA/iLA) to 19 (drug-coated balloon catheter in coronary vessels—DCB-CV). Most identified RCTs show high RoB. Only for five technologies, at least one RCT with low RoB was identified (Additional file 1, Appendix 9, Fig. S9.1).

The number of systematic reviews and HTA reports ranges from zero (e.g., flow-diverter in upper leg vessels—FD-ULV) to 37 (TAVI). The number of systematic reviews is higher than the number of RCTs for most technologies. For some technologies, there were several systematic reviews analyzing the same group of RCTs (e.g., intracranial endovascular mechanical thrombectomy—MT). More details on study characteristics can be found in Additional file 1, Appendix 9.

### Development of the body of evidence over time

In the first years of documented utilization, the number of available publications is low for almost every technology in the sample. Results from non-comparative study designs (LoE 4) usually dominate the picture. Despite the increasing share of evidence from LoE 1–3 over the course of the observation period (Additional file 1, Appendix 10, Fig. S10.1), the number of such publications remains beneath that of Level 4 for the majority of technologies (Additional file 1, Appendix 10, Fig. S10.2). A substantial lag (up to 9 years) can be observed between the first year of utilization and the publication of first results from RCTs for several technologies (Additional file 1, Appendix 9, Fig. S9.1).

### Results of the evidence on innovative technologies

**Fig. 3:**
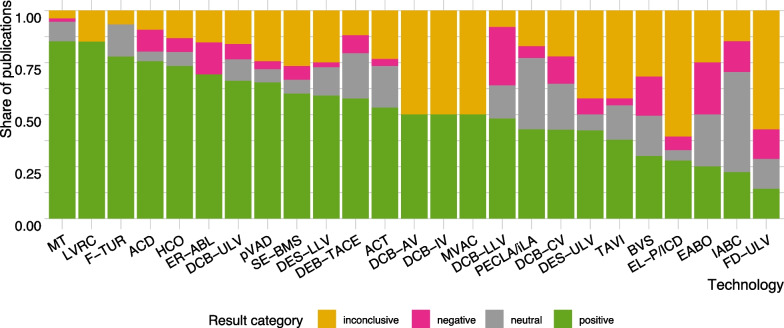
Conclusions of publications (LoE 1–4) per technology. Notes: The figure shows proportion of publications by results category on all identified publications per technology; Abbreviations are listed after the conclusion section, at the end of this article

Figure 3 shows the proportion of publications with positive, negative, neutral, and inconclusive results per technology. For some technologies, the share of inconclusive publications is relatively high; in particular case-series and other single-arm studies did not always fall into positive, negative, or neutral category due to ambiguity in the conclusions (e.g., better efficacy but poorer safety) (Additional file 1, Appendix 11, Fig. S11.1). A detailed distribution of publications by results category, LoE and technology is shown in Additional file 1, Appendix 11, Figs. S11.2, and 11.3 (A-C). Negative results tended to be observed more frequently among studies with a comparison group. On the contrary, LoE 4 studies tended to conclude positively more often.

### Utilization of innovative technologies

**Fig. 4:**
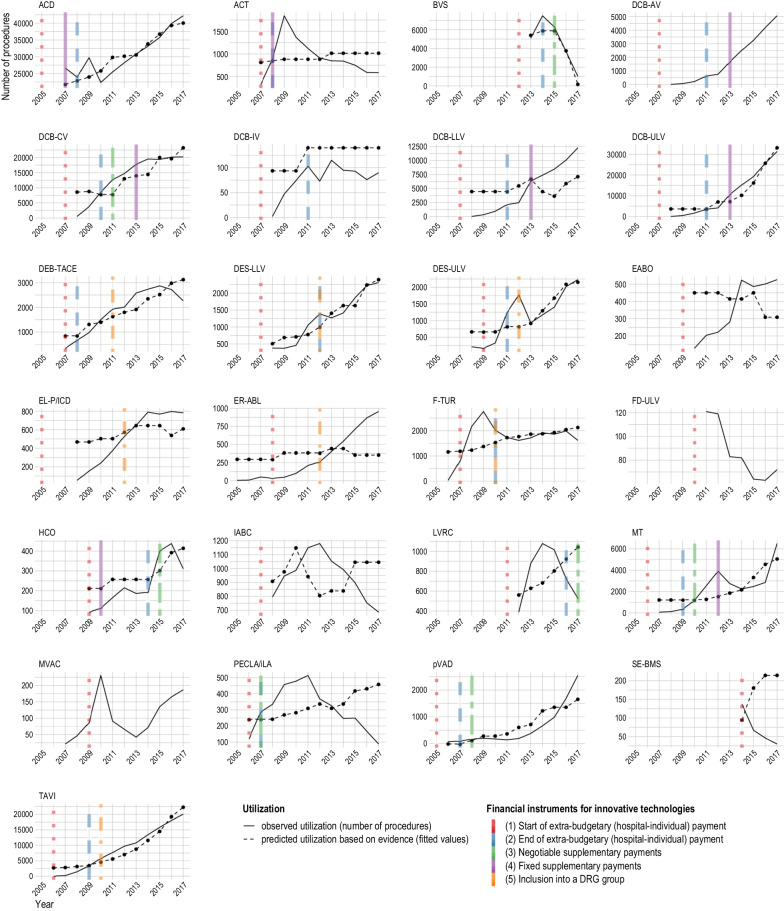
Relationship between utilization, evidence, and funding: Ability of LoE 1–3 evidence to predict utilization. Notes: Overlapping colored lines mean ending of one and beginning of another reimbursement type; Fitted values are based on results of evidence but not on financial instrument; Figures without fitted values represent technologies without evidence of LoE 1–3; For sources of information on financial instruments and number of hospital procedures see Additional file 1 Appendices 1 and 2; Abbreviations are listed after the conclusion section, at the end of this article.

The observed utilization of the technologies in the study sample, measured by billed number of procedures per year, is shown in Fig. 4. The maximum number of procedures per year ranges from 138 (2014, self-expanding bare metal stents in coronary vessels—SE-BMS) to 42,203 (2017, anticoagulation with citrate during dialysis—ACD). The number of years with reimbursed inpatient procedures identified through specific procedure codes in the observation period ranged from four (SE-BMS) to 13 (Cardiac event recorder after ablative measures for atrial fibrillation / atrial tachycardia—ER-ABL), with a median of 10 (2008).

### Funding of innovative technologies

As shown in Fig. 4, the beginning of utilization is linked to the permission to negotiate extra-budgetary payments with health insurance funds for most technologies. Exceptions are ER-ABL, F-TUR and mitral valve annuloplasty with clamp (MVAC), for which utilization starts earlier. For most technologies, the end of the permission to negotiate extra-budgetary payments coincides with the beginning of the next stage of reimbursement. At the end of the observation period, negotiable or fixed supplementary payments applied for 12 out of 25 technologies, while seven were included in a DRG. A change of reimbursement occurred after one to seven years. For the five remaining technologies (endo-aortic balloon occlusion with extracorporeal circulation—EABO, FD-ULV, bioactive coils for intracranial aneurysm therapy—IABC, MVAC, SE-BMS), funding did not change during the observation period.

### Relationship between utilization and other factors

The multilevel regression showed a statistically significant relationship between the direction of evidence and the direction of utilization (Table 1). The different orders of magnitude in utilization across technologies, resulting primarily from the varying prevalence of the underlying conditions, may explain the very high variance. The high intra-class correlation (ICC) confirms the appropriateness of the clustered approach [30, 32].

**Table 1:** Relationship between the available body of evidence and utilization: results of multilevel regression

	Number of hospital procedures		
Predictors	Estimates	95% CI	*p*-value
Fixed effects			
(Intercept)	2004.59	511.95–3517.92	0.0152^a^
Results of available body of evidence	67.80	14.09–117.98	0.0155^a^
Random effects			
σ^2^	3,092,340.71		
γ_00 technology_	11,621,363.15		
γ_11 technology. Results of available body of evidence_	10,344.00		
ρ_01 technology_	0.77		
ICC	0.95		
N _technology_	22		
Observations	217		
Marginal R^2^/Conditional R^2^	0.150/0.955		

Source: Created in Rstudio (Version 1.4.1717) using lme4 package; Note: ^a^* p* < .05

Figure 4 illustrates the agreement between observed annual utilization and predicted utilization based on evidence (fitted values) per technology. For most technologies, increasing utilization goes along with positive evidence (e.g., TAVI) or decreasing utilization goes along with negative evidence (e.g., BVS). In contrast, predicted values are not in line with observed values for seven technologies (e.g., Lung volume reduction by insertion of coils—LVRC); this implies that factors other than evidence had a stronger influence on utilization.

As can be seen in Fig. 4, no consistent patterns emerge regarding the relationship between trends in utilization and funding changes.

The impact of safety notifications and recalls is difficult to analyze, particularly as multiple products are available for most technologies in the sample. For technologies with at most two identified products and one identified recall (excimer laser extraction of pacemaker and defibrillator electrodes—EL-P/ICD [33] in 2012, dialysis with high cut-off dialysis membrane—HCO [34] in 2011, PECLA/iLA [35] in 2006), no relationship is observable. In the case of BVS, safety warnings restricting use to certain vessels [36] and to selected facilities participating in clinical registries [37] went along with negative evidence followed by a decline in utilization.

## Discussion

This work evaluated the relationship between the utilization of 25 technologies for different anatomical systems in German inpatient care and available clinical evidence, changes in funding and safety information.

The number of included publications per technology ranged from two to 498, with case-series and other non-comparative designs constituting the bulk of the evidence body. Although this reflects the interest of clinicians in sharing their experience with a certain technology in a real-world setting, such studies do not provide an adequate foundation to conclude on a technology’s comparative value. For seven of the 25 technologies, a maximum of one publication from either of the two highest evidence levels (systematic reviews of RCTs or individual RCTs) was identified despite high utilization numbers. In fact, for most included technologies there were few RCTs, predominantly with high RoB. A high volume of publications does not necessarily predicate the robustness of the evidence body on the benefit of a technology. Similarly, the availability of multiple systematic reviews of comparative studies (LoE 1a/2a) for the same technology does not necessarily result in information gain. For example, several systematic reviews/meta-analyses on stent retrievers for mechanical thrombectomy in acute stroke were identified in this work, published within two years, and combining the same six RCTs. What is more, findings in systematic reviews are only as robust as the studies included in the review allow.

The trend towards higher LoE over time was not always observable. For several technologies, a robust body of evidence (e.g., at least one RCT with low risk of bias) did not materialize for several years or even until the end of the observation period. Across technologies, the number of years and the number of procedures performed before adequate scientific evidence became available varied. The type of FDA approval, which is tied to post-market evidence generation requirements, and could thus have influenced the number and type of available studies [38, 39], was not investigated further. However, it appears that the lack of mandatory benefit assessment before reimbursement potentially exposed patients to undue harm (including lack of benefit) and the health system to inefficient spending. Other European countries, such as France [40] and the UK [41], have established pathways of comparative effectiveness assessment for new technologies prior to reimbursement. An evaluation pathway was established for certain high-risk innovative technologies in German inpatient care in 2016 (§137h SGB V), mandating hospitals seeking to negotiate extrabudgetary payments for the first time to provide the FJC with available evidence on effectiveness. This did not apply to any of the technologies in the sample.

A relationship between evidence and utilization could be shown for some technologies, but not all. Up to a point, this was to be expected, as there are many factors that influence the adoption of new technologies in health care organizations that could not be accounted for in the regression model. The available number of alternative technologies, operator experience, user-friendly operation, organizational culture, individual beliefs and preferences of operators as well as patient demand [42, 43] can play a role in the diffusion process. It is also thinkable that high initial acquisition costs lead to the continued use of technology despite the availability of better alternatives. Finally, even if a technology is reimbursable and has a positive evidence profile, insurers may not be willing to agree to extra-budgetary or supplementary payments that cover all costs.

The relationship between utilization and funding, as well as the relationship between utilization and safety notifications were explored in a qualitative manner, without clear results. However, this does not fully exclude the possibility that such relationships existed for any of the technologies. This study was not designed to predict what utilization might have looked like if funding had not changed over time from the most insecure (extra-budgetary payments) to more secure types of funding (e.g. adequate depiction in a DRG). For at least one technology, safety notices and restrictions could have strengthened the effect of clinical evidence, but this relationship also proved impossible to evaluate fully with this study design.

To ensure quality of care, it is important that reimbursed technologies are safe and effective. The first step towards achieving this is to have regulatory processes in place that only allow such medical devices to enter the market. The changes introduced by the MDR, which took effect in May 2021 [5, 8], could reduce the number of years of utilization without robust evidence in German inpatient care and elsewhere in Europe (and the time lag between CE-certification and FDA approval [44]) but this will also depend on how the regulation is implemented.

The second step is to assess the comparative effectiveness or even cost-effectiveness of new technologies prior to reimbursement. Every health system needs to balance timely access with certainty on the safety and effectiveness of an innovation, while distributing limited resources wisely. For new technologies that show promise but are not supported by adequate evidence yet, coverage with evidence development (CED) may provide a solution. CED is used by several European countries, such as Belgium, England, France, the Netherlands, Spain, and Switzerland [45], and has also been introduced in Germany [46]. It is important to design such programs carefully, particularly as public institutions are often not experienced in planning and conducting clinical trials, which can lead to delays and—depending on program set-up—challenges with access or inefficiencies [45]. Further, CED has the potential to support innovation from small and medium size manufacturers of medical devices, who may not be able to afford large clinical trials otherwise.

Moreover, it is important to equip CED programs with the mechanisms to stop reimbursement if the evidence is not sufficient or unfavorable. More broadly, implementing disinvestment approaches that effectively remove low-value technologies from health care provision is both crucial to enable optimal patient care and optimal resource utilization, and (politically) challenging to accomplish. While disinvestment strategies for pharmaceuticals in outpatient care are widely used in European countries [47], additional approaches are needed for disinvestment of medical devices in hospital care. Initiatives aiming to change clinical practice recommendations, such as “Choosing Wisely” and the “do not do” guideline of the National Institute for Health and Care Excellence (NICE) in England [48] could reduce the use of potentially ineffective or cost-ineffective devices. Removing potentially harmful medical devices from the health care market requires increased accountability provisions for manufacturers. With the introduction of the MDR, the EU has strengthened relevant provisions: it obliges manufacturers to continuously monitor the safety of marketed devices by establishing a post-market surveillance system for identifying and monitoring safety issues and implementing any necessary preventive and corrective actions (EU regulation 2017/745, Art. 83) [5]. However, removing a technology that is already established in clinical practice is usually faced with resistance from relevant stakeholders [49].

### Limitations

This work has several limitations. Although the sample of included technologies was selected in a systematic approach [22], it is not necessarily representative for all new technologies. Thus, these results remain indicative.

The calculation of utilization is based on procedure documentation available at RDC [21]. The validity of the data is limited by the quality of the underlying coding practices, which depends both on the experience of the coder and on the extent to which the codes used only capture the distinct technologies in the sample (as opposed to also capturing related technologies for which no unique codes existed yet). Furthermore, the procedure classification system is subject to regular changes, and codes may change over time as the classification becomes more detailed. This could have resulted in a distortion of observed utilization for some technologies (e.g., F-TUR).

Due to the rapid review methodology adopted to identify evidence, it is possible that either not all relevant citations were identified, or potentially relevant studies were excluded, e.g., through exclusion of languages other than German or English, and non-availability of full texts. Additional sources for clinical guidelines and HTA reports were chosen from the perspective of the German context; thus, these results are not exhaustive.

For feasibility reasons, results per publication were categorized based solely on the conclusions of the authors. This means that the consistency of these conclusions with outcomes reported in the publication results section was not investigated further and the potential influence of spin [50] has not been accounted for. Furthermore, studies with negative results are overall less likely to be published [51]—this study did not account for publication bias. Finally, different authors were involved in the selection and categorization of evidence per technology. Despite frequent meetings and consensus discussions with the full author team investigator bias cannot be excluded.

The aggregated variable representing the body of evidence in the multilevel regression model has the advantage of considering the entire body of evidence available up to the time point of utilization and avoiding distortions, for instance because of a single negative study. However, the disadvantage is that the impact of one single, crucial study, can be underestimated. Furthermore, the consideration and weighting of different study designs reflects a choice based on evidence hierarchies and could be subject to discussion. Finally, other observable (e.g., funding, safety warnings, disease incidence) and unobservable factors were not incorporated into the model.

## Conclusions

This is the first study investigating the relationship between evidence and utilization for a sample of 25 new medical technologies in German inpatient care in a descriptive and empirical manner. The body of clinical evidence per technology often consisted mainly of non-comparative studies; its robustness increased over time for many but not all technologies. A relationship between evidence and utilization could be shown for some, albeit not all, technologies. The influence of funding and safety notices requires further investigation. These results reveal that a re-evaluation of market approval standards and HTA pathways might be warranted.

### Supplementary Information

**Additional file 1: Appendix 1.** Description of included technologies;** Appendix 2.** Searched sources for evidence;** Appendix 3.** Inclusion criteria of publications;** Appendix 4.** Screening of records obtained from registries;** Appendix 5.** Data extraction;** Appendix 6.** Risk of bias assessment of RCTs;** Appendix 7.** Description of the regression model;** Appendix 8.** Results of the search for evidence per database and screening step;** Appendix 9.** Characteristics of included studies;** Appendix 10.** Development of body of evidence;** Appendix 11.** Results of evidence for technologies in sample;** Appendix 12.** Recommendations from clinical guidelines;** Appendix 13.** References.

## Abbreviations

ACD: Anticoagulation with citrate during dialysis
ACT: Adjustable continence therapy
BVS: Bioresorbable Vascular Scaffold in coronary vessels
CED: Coverage with evidence development
DCB-AV: Drug-coated balloon catheter in abdominal vessels
DCB-CV: Drug-coated balloon catheter in coronary vessels
DCB-IV: Drug-coated balloon catheter in intracranial vessels
DCB-LLV: Drug-coated balloon catheter in lower leg vessels
DCB-ULV: Drug-coated balloon catheter in upper leg vessels
DEB-TACE: Drug-eluting beads for trans-arterial chemoembolization
DES-LLV: Implantation of a drug-eluting stent in lower leg vessels
DES-ULV: Implantation of a drug-eluting stent in upper leg vessels
DRG: Diagnosis-related groups
EABO: Endo-aortic balloon occlusion with extracorporeal circulation
EL-P/ICD: Excimer laser extraction of pacemaker and defibrillator electrodes
ER-ABL: Cardiac event recorder after ablative measures for atrial fibrillation/atrial tachycardia
EU: European Union
FDA: Food and Drug Administration
FD-ULV: Flow-diverter (Hemodynamically effective implant for endovascular treatment of peripheral aneurysms) in upper leg vessels
FJC: Federal Joint Committee (Gemeinsamer Bundesausschuss)
F-TUR: Fluorescence-assisted transurethral resection
HCO: Dialysis with high cut-off dialysis membrane
HTA: Health technology assessment
IABC: Bioactive coils for intracranial aneurysm therapy
InEK: Institute for the Hospital Remuneration System (Institut fuer das Entgeltsystem im Krankenhaus)
IQWiG: Institute for Quality and Efficiency in Health Care (Institut für Qualität und Wirtschaftlichkeit im Gesundheitswesen)
LVRC: Lung volume reduction by insertion of coils
LoE: Level of evidence
MDR: Medical Device Regulation
MDS: National Association of Health Insurance Funds (Medizinischer Dienst des Spitzenverbandes Bund der Krankenkassen e.V.)
MT: Intracranial endovascular mechanical thrombectomy
MVAC: Mitral valve annuloplasty with clamp
NUB: New Diagnostic and Treatment Methods (Neue Untersuchungs- und Behandlungsmethode)
OPS: German Procedures Classification (Operationen- und Prozedurenschluessel)
PECLA/ iLA: Pumpless Extracorporeal Lung Assist/Interventional Lung Assist
pVAD: Percutaneous ventricular assist device (Micro-axial blood pump)
RCT: Randomized controlled clinical trial
RDC: Research Data Centre
RoB: Risk of bias
SE-BMS: Self-expanding bare metal stents in coronary vessels
SGB V: German Social Code Book V
TAVI: Transcatheter aortic valve implantation
US: United States

## Declarations

### Availability of data and materials

The datasets used and/or analysed during the current study are available from the corresponding author on reasonable request.
