# Supplementary material for: Utilization of innovative medical technologies in German inpatient care: does evidence matter?
Source: Health Res Policy Syst. 2023 Oct 2;21:100. doi: 10.1186/s12961-023-01047-w (PMC10546629; doi:10.1186/s12961-023-01047-w)
Supplement: Supplementary file 1 — Additional file 1: Appendix 1. Description of included technologies; Appendix 2. Searched sources for evidence; Appendix 3. Inclusion criteria of publications; Appendix 4. Screening of records obtained from registries; Appendix 5. Data extraction; Appendix 6. Risk of bias assessment of RCTs; Appendix 7. Description of the regression model; Appendix 8. Results of the search for evidence per database and screening step; Appendix 9. Characteristics of included studies; Appendix 10. Development of body of evidence; Appendix 11. Results of evidence for technologies in sample; Appendix 12. Recommendations from clinical guidelines; Appendix 13. References. [file 12961_2023_1047_MOESM1_ESM.pdf]

Additional file 1: Utilization of Innovative Medical Technologies in German Inpatient Care: Does Evidence Matter?

Table of contents

Table of contents 1
Table of abbreviations 3
Appendix 1: Description of included technologies 4
Table 1.1 Description of technologies and respective procedure codes (alphabetically sorted) ... 4
Table 1.2 Technologies, products and year of CE marking sorted by anatomical region 7
Appendix 2: Searched sources for evidence 10
Table 2.1 Sources of evidence 10
Appendix 3: Inclusion criteria of publications 11
Table 3.1 General inclusion and exclusion criteria 12
Figure 3.1 Classification of evidence - excerpt of proceeding rules by G-BA 2. chapter § 11 (3) [4, 5] 13
Appendix 4: Screening of records obtained from registries 14
Appendix 5: Data extraction 15
Table 5.1 Extracted information per publication (individual study) 16
Table 5.2 Extracted information per publication (systematic review, meta-analysis, HTA) 17
Table 5.3 Extracted information per guideline 17
Appendix 6: Risk of bias (RoB) assessment of RCTs 18
Table 6.1 Risk of bias (RoB) tool used for RoB assessment 18
Appendix 7: Description of the regression model 19
Description of the variable with weighted cumulative evidence – the "new variable" 19
Derivation of the regression model 20
Appendix 8: Results of the search for evidence per database and screening step 21
Table 8.1 Results of the search for evidence (individual studies, systematic review, meta-analysis, HTA) 21
Table 8.2 Results of the search for guidelines, HTA, safety notifications and in clinical trial registers 22
Appendix 9: Characteristics of included studies 23
Figure 9.0.1 Randomized controlled trials and results of Risk of Bias (RoB) assessment 23
Table 9.1 Number of included publications with systematic reviews and randomized controlled trials 24

Appendix 10: Development of body of evidence 25

Figure 10.1 Evidence development: Share of publications of LoE 1-3 studies on all publications and its trend of grow by anatomical system and technology 25

Figure 10.2 Evidence development: Increase of accumulated number of publications LoE 1-3 and LoE 4 studies by anatomic system and technology 26

Appendix 11: Results of evidence for technologies in sample 27

Figure 11.1 Negative or inconclusive results in publications by LoE and year of publication based on all technologies of sample 27

Figure 11.2 All categories of results in publications by LoE and year of publication based on all technologies of sample 27

Figure 11.3 (A-C) Results in publications presented by LoE, year of publication and technology (by availability of negative results) 28

Appendix 12: Recommendations from clinical guidelines 32

Figure 12.1 Recommendations from identified clinical guidelines 32

Appendix 13: References 33

References 33

Table of abbreviations

ACD	Anticoagulation with citrate during dialysis
ACT	Adjustable continence therapy
BVS	Bioresorbable Vascular Scaffold in coronary vessels
BfArM	Federal Institute for Drugs and Medical Devices (*Bundesinstitut für Arzneimittel und Medizinprodukte*)
DCB-AV	Drug-coated balloon catheter in abdominal vessels
DCB-CV	Drug-coated balloon catheter in coronary vessels
DCB-IV	Drug-coated balloon catheter in intracranial vessels
DCB-LLV	Drug-coated balloon catheter in lower leg vessels
DCB-ULV	Drug-coated balloon catheter in upper leg vessels
DEB-TACE	Drug-eluting beads for transarterial chemoembolization
DES-LLV	Implantation of a drug-eluting stent in lower leg vessels
DES-ULV	Implantation of a drug-eluting stent in upper leg vessels
DRG	Diagnosis-Related Groups
EABO	Endoaortic balloon occlusion with extracorporeal circulation
EL-P/ ICD	Excimer laser extraction of pacemaker and defibrillator electrodes
ER-ABL	Cardiac event recorder after ablative measures for atrial fibrillation / atrial tachycardia
FD-ULV	Flow-diverter (Hemodynamically effective implant for endovascular treatment of peripheral aneurysms) in upper leg vessels
F-TUR	Fluorescence-assisted transurethral resection
G-BA	Federal Joint Committee (*Gemeinsamer Bundesausschuss)*
HCO	Dialysis with high cut-off dialysis membrane
IABC	Bioactive coils for intracranial aneurysm therapy
InEK	Institute for the Hospital Remuneration System (*Institut fuer das Entgeltsystem im Krankenhaus*)
IQWiG	Institute for Quality and Efficiency in Health Care (*Institut für Qualität und Wirtschaftlichkeit im Gesundheitswesen*)
LVRC	Lung volume reduction by insertion of coils
MDS	National Association of Health Insurance Funds (Medizinischer Dienst des Spitzenverbandes Bund der Krankenkassen e.V.)
MT	Intracranial endovascular mechanical thrombectomy
MVAC	Mitral valve annuloplasty with clamp
NUB	*New Diagnostic and Treatment Methods (Neue Untersuchungs- und Behandlungsmethode)*
OPS	German Procedures Classification (*Operationen- und Prozedurenschluessel*)
PECLA/ iLA	Pumpless Extracorporeal Lung Assist/ Interventional Lung Assist
pVAD	Percutaneous ventricular assist device (Microaxial blood pump)
RCT	Randomized controlled trial
RoB	Risk of bias
SE-BMS	Self-expanding bare metal stents in coronary vessels
TAVI	Transcatheter aortic valve implantation

Appendix 1: Description of included technologies

From the total sample of 27 technologies [1] 25 were included into the analysis. Of these, 19 are used for diseases of the circulatory system, four for diseases of the urogenital system, and two for respiratory conditions.

Table 1.1 Description of technologies and respective procedure codes (alphabetically sorted)

Abbreviation of NUB (N=25)	Area of application	Description	Hospital procedures: Min (year)/ Max (year)[1]	Procedure codes[2]
ACD	Citrate anticoagulation as a heparin substitute during renal replacement therapy for acute kidney injury or chronic kidney disease	Extracorporeal renal replacement therapy with substitution of heparin with citrate, usually in cases of heparin intolerance or risk of bleeding	22336 (2010) / 42203 (2017)	8-854.3#, 8-854.5#, 8-854.7#, 8-855.4#, 8-855.6#, 8-855.8#
ACT	Treatment of severe urinary incontinence	The fully implantable fluid-filled silicone balloons are placed on both sides of the urethra at the bladder outlet for the treatment of urinary incontinence in men and women.	327 (2007) / 1843 (2009)	5-596.7#
BVS	Treatment of coronary heart disease (CHD)	Implant is inserted endovascularly into the coronary vessel and keeps the stenosed lesion mechanically open for a period of several years, during which time it is completely reabsorbed by the body.	1016 (2017) / 7494 (2014)	8-83d0#
DCB-CV	Treatment of CHD	Further development of a conventional balloon catheter: the surface is coated with an antiproliferative drug which is released immediately at the site of vasoconstriction and is intended to counteract restenosis	533 (2008) / 20220 (2017)	8-837.0# in connection to 8-83b.b* (*= 0, 2-9)
DCB-AV	Treatment of PAD in abdominal vessels		3 (2008) / 5061 (2017)	8-836.09 [before 2017] or 8-836.0q, 8-836.0r [in 2017] in connection to 8-83b.b* (*= 0, 2-9, a-d)
DCB-IV	Treatment of PAD in intracranial vessels		2 (2008) / 115 (2013)	8-836.00 in connection to 8-83b.b* (*= 0, 2-9, a-d)
DCB-LLV	Treatment of PAD in lower leg vessels		26 (2008) / 12281 (2017)	8-836.0c in connection to 8-83b.b*(*= 0, 2-9, a-d)
DCB-ULV	Treatment of PAD in upper leg vessels		13 (2008) / 31350 (2017)	8-836.0b [before 2017] or 8-836.0s, 8-836.0t [in 2017] in connection to 8-83b.b*(*= 0, 2-9, a-d)
DEB-TACE	Treatment of hepatocellular carcinoma	Hydrogel microspheres made of polyvinyl alcohol that can be loaded with doxorubicin. They are used for transarterial chemoembolization of vessels supplying malignant tumors, delivering a local, sustained dose of doxorubicin.	345 (2007) / 2870 (2015)	8-836.k in connection to 8-83b.10

Abbreviation of NUB (N=25)	Area of application	Description	Hospital procedures: Min (year)/ Max (year)[1]	Procedure codes[2]
DES-LLV	Treatment of peripheral arterial occlusive disease (PAD) in lower leg vessels	Stents coated with cytostatic drugs (paclitaxel) or immunosuppressants (sirolimus, everolimus) (so-called drug eluting stents, DES); the coating is intended to reduce the risk of restenosis.	374 (2009) / 2310 (2017)	8-841.0c, 8-841.1c, 8-841.2c, 8-841.3c, 8-841.4c, 8-841.5c
DES-ULV	Treatment of PAD in upper leg vessels	Stents coated with cytostatic drugs (paclitaxel) or immunosuppressants (sirolimus, everolimus) (so-called drug eluting stents, DES); the coating is intended to reduce the risk of restenosis.	166 (2009) / 2230 (2017)	8-841.0*, 8-841.1*, 8-841.2*, 8-841.3*, 8-841.4*, 8-841.5* (*= b [before 2017], s or t [in 2017])
EABO	Cardiac surgery without thoracotomy	Closure of the ascending aorta by balloon occlusion from the inside (endo-clamping), arrest of the heart by intra-aortic balloon occlusion cannulation.	129 (2010) / 526 (2017)	8-851.01, 8-851.11, 8-851.31, 8-851.41, 8-851.51.
EL-P/ICD	Removal of defibrillator or pacemaker electrodes from the heart if indicated (e.g., infection or malfunction)	Circularly arranged optical fibers with an inner lumen that can accommodate the pacing electrode. A device is advanced via an electrode lead to the electrode tip located in the heart, the excimer laser emits short, high energy, low temperature pulses that ablate small amounts of tissue (photoablation) and expose the electrode.	54 (2008) / 797 (2016)	5-378.a0 in connection to 5-378.1#, 5-378.2#, 5-378.6#, 5-378.7#
ER-ABL	Diagnosis of cardiac arrhythmias (tachyarrhythmia, atrial fibrillation) after ablative procedures	Device for continuous monitoring of ECG information over a long period of time, which is implanted subcutaneously on an outpatient basis under local anaesthesia. A single-channel ECG is continuously recorded in an endless loop („loop recorder") via surface electrodes on the housing. Pathological sequences can be automatically detected and stored.	5 (2005) / 956 (2017)	5-377.8 in connection to 8-835.##
FD-ULV	Treatment of aneurysm of upper leg vessels	Self-expanding blood flow modulators (grid/stent structures) which are placed endovascularly directly into the aneurysm outpouching so that a functional separation between the outflow vessel and the aneurysm outpouching is created at the aneurysm base.	63 (2016) / 121 (2011)	8-84b.0*, 8-84b.1*, 8-84b.2*, 8-84b.3*, 8-84b.4*, 8-84b.5* (*= b [before 2017], s or t [in 2017])
F-TUR	Diagnosis of (non-muscle invasive) bladder carcinoma or carcinoma in situ or lesions of unclear origin of the bladder and resection	Enhancement of the visual contrast between benign tissue and tumor cells by inducing a photodynamic process leading to the selective fluorescence emission of tumor cells	32 (2006) / 2763 (2009)	OPS 5-573.41 or 1-661 in connection to 5-989
HCO	Treatment of acute kidney injury caused by low weight molecules	Dialysis membrane with the aim to selectively remove low weight molecules (molecular weight between 15-60 kDa, such as light chains in multiple myeloma).	92 (2009) / 438 (2016)	8-854.8
IABC	Treatment of intracranial aneurysms	Coils endovascularly inserted with a microcatheter and coated with bioactive materials (polyglycolic acid, polylactic acid) to better seal the aneurysm.	686 (2017) / 1180 (2012)	8-836.m0 in connection to 8-83b.31

Abbreviation of NUB (N=25)	Area of application	Description	Hospital procedures: Min (year)/ Max (year)[1]	Procedure codes[2]
LVRC	Treatment of lung emphysema	Lung volume reduction through the bronchoscopic use of spirals (coils), which are applied stretched and after release take their predetermined shape and pull the lung parenchyma along.	388 (2012) / 1079 (2014)	5-339.8
MT	Treatment of acute ischemic stroke (intracranial)	Retriever systems inserted endovascularly into the affected vessel, which mechanically remove thrombi	74 (2007) / 6499 (2017)	8-836.80 in connection to 8-83b.80, 8-83b.82, 8-83b.83; 8-836.80 in connection 8-83b.84, 8-83b.85, 8-83b.86
MVAC	Treatment of mitral valve regurgitation	Endovascular indirect tightening of the connective tissue of the mitral valve annulus to bring dehiscent mitral valve leaflets closer together and reduce insufficiency.	21 (2007) / 231 (2010)	5-35a.2
PECLA/iLA	Treatment of acute respiratory distress syndrome	System for pumpless support of lung function for emergency and intensive care medicine. Carbon dioxide is removed from arterial blood and oxygen is added by membrane diffusion.	88 (2017) / 514 (2011)	8-852.2#
pVAD	Acute heart failure, cardiogenic shock, or cardiovascular support during PCI	Minimally invasive heart support system (left ventricle) that can be introduced into the heart via a percutaneous access in the groin via the femoral artery without opening the chest and can also be removed in the same way.	72 (2006) / 2546 (2017)	8-839.42, 8-839.43
SE-BMS	Treatment of CHD	Stents made of nitinol, which have a memory effect in the sense of shape memory, and negative recoil. This can mean additional lumen gain in the stented vessel segment over the course of days and weeks after coronary intervention.	30 (2017) / 138 (2014)	8-83d.1#
TAVI	Treatment of valvular insufficiency or stenosis of the aortic valve	Minimally invasive replacement of heart valves, which exposes the patient to less intraoperative stress. The new valve is placed in position via a catheter and deployed on site.	68 (2006) / 20021 (2017)	5-35a.0#

Notes: [1]Data was provided by the funder, available at [2]; [2]All procedure codes from the OPS version 2005 to the OPS version 2017 can be accessed on the pages of the Federal Institute for Drugs and Medical Devices (BfArM), formerly the German Institute of Medical Documentation and Information (DIMDI) [3]; **#** The „#“ symbol replaces the 5th or 6th digit in the procedure code, indicating that the codes with these positions have been included in the calculation of procedure numbers. The „#“ represents any number or letter within the specified root, as defined in the procedure catalogue.

Abbreviations: ACD - Anticoagulation with citrate during dialysis; ACT - Adjustable continence therapy; BVS - Bioresorbable Vascular Scaffold in coronary vessels; DCB-AV - Drug-coated balloon catheter in abdominal vessels; DCB-CV - Drug-coated balloon catheter in coronary vessels; DCB-IV - Drug-coated balloon catheter in intracranial vessels; DCB-LLV - Drug-coated balloon catheter in lower leg vessels; DCB-ULV - Drug-coated balloon catheter in upper leg vessels; DEB-TACE - Drug-eluting beads for trans-arterial chemoembolization; DES-LLV - Implantation of a drug-eluting stent in lower leg vessels; DES-ULV - Implantation of a drug-eluting stent in upper leg vessels; EABO – Endo-aortic balloon occlusion with extracorporeal circulation; EL-P/ ICD - Excimer laser extraction of pacemaker and defibrillator electrodes; ER-ABL - Cardiac event recorder after ablative measures for atrial fibrillation / atrial tachycardia; FD-ULV - Flow-diverter (Hemodynamically effective implant for endovascular treatment of peripheral aneurysms) in upper leg vessels; F-TUR - Fluorescence-assisted transurethral resection; HCO - Dialysis with high cut-off dialysis membrane; IABC - Bioactive coils for intracranial aneurysm therapy; LVRC - Lung volume reduction by insertion of coils; MT - Intracranial endovascular mechanical thrombectomy; MVAC - Mitral valve annuloplasty with clamp; NUB – New examination and treatment method / Neue Untersuchungs- und Behandlungsmethode; PECLA/ iLA - Pumpless Extracorporeal Lung Assist/ Interventional Lung Assist; pVAD - Percutaneous ventricular assist device (Micro-axial blood pump); SE-BMS - Self-expanding bare metal stents in coronary vessels; TAVI - Transcatheter aortic valve implantation

Across the 25 included technologies, the number of identified products available in Europe and Germany between 2005 and 2017 ranges from one (e.g., MVAC) to 16 (MT) (Table 1.2). Most products were CE-certified for the first time after 2000. For some technologies, it was not possible to determine a product intended specifically for the indication (i.e., DCB-AV, DCB-IV, and DES-LLV), and for some products the year of approval was unavailable.

Table 1.2 Technologies, products and year of CE marking sorted by anatomical region

Table 1 .2 Technologies in sample by anatomical region

Anatomical regions of procedures			Procedure	Abb	Product (Manufacturer)	CE-Mark year
Respiratory system			Lung volume reduction by insertion of coils	LVRC	ELEVAIR Endobronchial Coil System (PneuRX, now Boston Scientific)	2010
			Pumpless Extracorporeal Lung Assist/ Interventional Lung Assist	PECLA/ iLA	Novalung iLA (Novalung)	2002
Cardiovascular system	Cardiac procedures	Ventricular	Transcatheter aortic valve implantation	TAVI	SAPIEN (Edwards Lifesciences)	2007
					CoreVALVE (Medtronic Inc.)	2007
					Acurate (Symetis Inc.)	2011
					JenaValve TA/ neo TF (Symetis Inc.)	2011
					Direct Flow Medical (Direct Flow Medical, Inc.)	2013
					Lotus (Boston Scientific)	2013
					Portico (St. Jude Medical)	2015
					Centera (Edwards Lifesciences)	2017
					Allegra (New Valve Technology, now Biosensors)	2017
			Mitral valve annuloplasty with clamp	MVAC	CARILLON™ Mitral Contour System (Cardiac Dimensions)	2009
			Percutaneous ventricular assist device (Microaxial blood pump)	pVAD	Impella 5.0 (Abiomed)	2001
					Impella 2.5 (Abiomed)	2010
					Impella CP (Abiomed)	2014
					Impella RP (Abiomed)	2014
					HeartMate PHP (Abbott)	2015
		Other cardiac	Cardiac event recorder after ablative measures for atrial fibrillation / atrial tachycardia	ER-ABL	Reveal Plus/Reveal XT/Reveal DX (Medtronic)	2007
					BioMonitor I (BIOTRONIK)	2012
					Reveal LinQ (Medtronic Inc)	2014
					BioMonitor II (BIOTRONIK)	2015
					Confirm Rx (Abbott)	2017
			Excimer laser extraction of pacemaker and defibrillator electrodes	EL-P/ ICD	SLS I (Spectranetics)	1994
	Endovascular procedures	Intracranial	Drug-coated balloon catheter in intracranial vessels	DCB-IV	none (DCB for coronary vessels)	n.a.
			Bioactive coils for intracranial aneurysm therapy	IABC	Cerecyte (Micrus Endovascular)	2005
					Axium MicroFX (ev3/Medtronic)	2007
					Matrix 1. & 2. gen (Boston Scientific)	n.a.
					Nexu (ev3/Covidien/Micro Therapeutics/ Medtronic)	n.a.
			Intracranial endovascular mechanical thrombectomy	MT	Merci™ Retriever System (Concentric Medical, now Stryker)	2002
					Catch™-Retriever (Balt)	2004
					Phenox Clot Retriever (Phenox, Bochum, Deutschland)	2006
					Amplatz Goose-Neck™ (Microvena)	n.a.
					Attracter-18™ (Boston Scientific)	n.a.
					Distal Access Catherer (DAC™, initial Concentric Medical, now Stryker)	n.a.
					Catch Plus (Balt)	2005

Table 1 .2 Technologies in sample by anatomical region

Anatomical regions of procedures	Procedure	Abb	Product (Manufacturer)	CE-Mark year
			Solitaire™ (Micro Therapeutics, now Medtronic)	2009
			Trevo™ (Concentric Medical, now Stryker)	2010
			Revive (Codman Neuro Johnson&Johnson)	2011
			pREset (Phenox)	2011
			Mind Frame Capture LP (Mindframe, now Medtronic)	2011
			APERIO ™	2012
			Embotrap (Neuravi, now Cerenovus Johnson&Johnson)	2013
			BONnet (Phenox)	2013
			Eric (Microvention)	2014
Thorocal	Endo-aortic balloon occlusion with extracorporeal circulation	EABO	Heartport (Heartport, now Edwards Lifesciences)	1997
			Estech arterial RAP (ESTECH)	1999
			EndoClamp (CardioVations, Ethicon Inc., Johnson & Johnson, now Edwards Lifesciences)	2012
			IntraClude (Edwards Lifesciences)	2012
Coronary	Bioresorbable Vascular Scaffold in coronary vessels	BVS	Absorb (Abbott)	2010
			DESolve (Elixir Medical)	2014
			Magmaris (Biotronik)	2016
			Fantom (REVA Medical, Inc.)	2017
	Self-expanding bare metal stents in coronary vessels	SE-BMS	Radius (Scimed Life Systems, Inc, now Boston Scientific)	2000*
			Cappella Sideguard (Cappella)	2009
			vProtect Luminal Shield (Prescient Medical)	2009
			CardioMind Sparrow (CardioMind)	2010
			Stentys (Stentys SA)	2010
	Drug-coated balloon catheter in coronary vessels	DCB-CV	Dior PCB (Eurocor)	2007
			Sequent Please Neo (B.Braun Melsungen)	2009
			Elutax SV (Aachen Resonance)	2013
			Restore DEB (Cardionovum)	n.a.
Abd.	Drug-coated balloon catheter in abdominal vessels	DCB-AV	none (DCB with experimental drug coating)	n.a.
Peripheral	Flow-diverter (Hemodynamically effective implant for endovascular treatment of peripheral aneurysms) in upper leg vessels	FD-ULV	Peripheral Multilayer Flow Modulator (Cardiatis SA)	2009
	Drug-coated balloon catheter in upper leg vessels	DCB-ULV	SeQuent Please OTW (B.Braun Melsungen)	2009
			IN.PACT Admiral (Medtronic/Invatec)	2010
			Advance PTX (Cook Medical)	2011
			Cotavance Balloon (Medrad/Bayer)	2011
			Legflow RX/OTW (Cardinovum)	2011
			Lutonix (CR Bard)	2012
			Elutax SV (Aachen Resonances/Abmedica)	2013
			Ranger (Boston Scientific)	2014
			Luminor (iVascular)	2016
			FREEWAY (Eurocor)	2016
			Stellarex (Covidien/ Spectranetics/ Philips)	2016
			Passeo-18 Lux (Biotronik)	2016
	Implantation of a drug-eluting stent in upper leg vessels	DES-ULV	Zilver PTX (Cook Medical)	2009
			Eluvia (Boston Scientific)	2016
	Drug-coated balloon catheter in lower leg vessels	DCB-LLV	Legflow RX/OTW (Cardinovum)	2011
			Agent Drug-Coated Balloon (Bosten Scientific)	2014

Table 1 .2 Technologies in sample by anatomical region				
**Anatomical regions of procedures**	**Procedure**	**Abb**	**Product (Manufacturer)**	**CE-Mark year**
	Implantation of a drug-eluting stent in lower leg vessels	DES-LLV	none (DES for coronary vessels)	n.a.
Urinary system	Adjustable continence therapy	ACT	ACT/ProACT (Uromedica)	2002
	Fluorescence-assisted transurethral resection	F-TUR	5-Aminolevulinic acid, Levulan (DUSA Pharm.)	1990s **
			KarlStorz D-Light C PDD System	1998
			Hexaminolevulinate, Hexvix® (Photocure)	2005
	Anticoagulation with citrate during dialysis	ACD	Sodium Citrate	1990s **
	Dialysis with high cut-off dialysis (HCO) membrane	HCO	HCO-Membrane Gambro Theralite (Gambro Dialysatoren, now Baxter)	2007
Antineoplastic	Drug-eluting beads for transarterial chemoembolization	DEB-TACE	DC Beads (Biocompatibles UK, now Boston Scientific)	2003
			HepaSphere (Merit Medical)	2007
			Embozene Tandem (Boston Scientific)	2012
			LifePearl (Terumo)	2015
			DC Beads Lumi (Boston Scientific)	2017

Notes: * approval year of the first product variation; ** exact year of approval not available;
Abbreviations: Abd – abdominal; DCB – drig coated baloons; DES – drug eluting stents; PDD - photodynamic diagnostics; UK – United Kingdom
Source: Compiled by the authors based on different sources

Appendix 2: Searched sources for evidence

Table 2.1 Sources of evidence

Sources of information used in the search for evidence	Date of the search
**Databases**	
PubMed (via PubMed)<br>Ovid MEDLINE(R) ALL 1946 to May 2019<br>Embase 1980 to 2019 May (via Ovid)<br>Cochrane Library (Cochrane Reviews, Trials)	May 2019 (with single exclusions)
**HTA**	
LBI-HTA (http://eprints.hta.lbg.ac.at/)	Apr-May 2019
IQWiG (https://www.iqwig.de/de/projekte-ergebnisse/publikationen/iqwig-berichte.1071.html)	Mar 2019
DIMDI-DAHTA (https://www.dimdi.de/dynamic/de/weitere-fachdienste/health-technology-assessment/)	Mar 2019
CRD/INAHTA (https://www.crd.york.ac.uk/CRDWeb/)	Apr-Jun 2019
EUnetHTA (https://www.eunethta.eu/assessment-archive-2006-2015/ ; https://www.eunethta.eu/rapid-reas/)	Apr 19
Gemeinsamer Bundesausschuss (G-BA) (https://www.g-ba.de/beschluesse)	Sep 19
Publicly availably MDS reports (Google)	Sep 19
**Clinical trial registries**	
International clinical trials registry platform (http://apps.who.int/trialsearch/)<br>ClinicalTrials.gov (https://clinicaltrials.gov/)	2019-2020
**Clinical guidelines**	
AWMF (https://www.awmf.org/leitlinien/aktuelle-leitlinien.html)	Apr 19
European Society of Cardiology (ESC) (https://www.escardio.org/Guidelines)<br>European Society of Thoracic Surgeons (ESTS) (http://www.ests.org/guidelines_and_evidence/guideline_database.aspx)<br>European Association of Urology (EAU) (https://uroweb.org/individual-guidelines/oncology-guidelines/)<br>European Society of Minimally Invasive Neurological Therapy (ESMINT) (https://www.esmint.eu/)<br>European Society for Medical Oncology (ESMO) (https://www.esmo.org/Guidelines)<br>European Society for Vascular Surgery (ESVS) (http://www.esvs.org/journal/guidelines/)<br>European Society for Vascular Medicine (ESVM) (http://vascular-medicine.org/guidelines/)<br>European Academy of Neurology (EAN) (https://www.ean.org/Neurology-Guidelines.2680.0.html)<br>European Respiratory Society (ERS) (https://www.ers-education.org/guidelines.aspx)<br>European Stroke Organisation (ESO) (https://eso-stroke.org/guidelines/eso-guideline-directory/#acute-stroke)	Jul-Aug 2019
**Safety notifications**	
Bundesinstitut für Arzneimittel und Medizinprodukte (BfArM) (https://www.bfarm.de/)<br>The Implant Files (https://medicaldevices.icij.org/)	2019-2020 (2021*)
**Information on funding**	
**Diagnosis-related groups (DRG) and supplementary payments in 2005-2017** retrieved from https://www.g-drg.de/Archiv: DRG classification 2005 to DRG classification **„Neue Untersuchungs- und Behandlungsmethoden“ (NUB), lists from 2005-2017** retrieved from https://www.g-drg.de/Archiv: §6 Abs. 2 KHEntgG)	Jul-Aug 2019

Source: Compiled by the authors; Notes: *last search was conducted in 2021.

Appendix 3: Inclusion criteria of publications

General inclusion criteria of publications applied in systematic literature search and selection are presented in table 2.2. We included publications:

- of human studies investigating the selected technology as intervention or comparator for the indication described by procedure codes
- of primary and secondary studies with level of evidence (LoE) 1 to 4 followed to definition of 2. chapter §11 (3) by proceedings rules of the *G-BA [4, 5]* and described below
- published as a journal article or as grey literature two years before the documentation of the first hospital utilization (at the earliest in 2003) and up to 2017 with available full text
- in English or German language.

No strict limitations were specified for the comparator intervention and endpoints. Studies reporting only cost data, with no possibility for inference of the benefit of a technology, were excluded. To ensure consistency to the utilization curves, we introduced a 10% rule: Studies were included into the analysis if the proportion of patients with deviation from the intended intervention in one study arm did not exceed 10 %. Based on the assumption that every type of a study could have influenced the utilization, comparative studies that investigated different versions of the same product under the same technology would not be excluded but included as a non-comparative (LoE 4) study. The general inclusion and exclusion criteria were adjusted and clarified during the processing of the first few technologies. The technology specific inclusion and exclusion criteria are available upon request.

For systematic reviews and meta-analyses, it was required that the publication identifies oneself in the title, abstract, introduction or methods as a systematic review or Meta-analysis and the methodology was sufficiently described to demonstrate a systematic approach, i.e., methods of literature search and selection were reported.

Health technology assessment (HTA) reports were included only if they explicitly investigated the benefit of one of the technologies in sample. No strict limitations were defined with regard to comparative interventions.

Clinical guidelines were included if they were published by national (Germany, Austria, Switzerland) and supranational professional societies between 2005 and 2017 and contained specific recommendations for or against the use of the technology in sample (see table 2.1 for sources of information).

Table 3.1 General inclusion and exclusion criteria[1]

pico	*Inclusion criteria*[2]	*Exclusion criteria*
**P**	a) The population of interest (adults and/or children)<br>b) Indication of interest<br>c) Subgroups with the same indication	a) In-vitro; experimental models; animals<br>b) Indication is not within the definition<br>c) Subgroups of population after treatment (e.g., with complications)
**I**	The main aim of the publication is to evaluate effects of the technology captured by the OPS (the intervention within the PICO of the publication is the technology of interest, in some cases the technology of interest can be a comparator)<br>a) There should be at least one arm in the evaluation which include **only the** technology of interest with similar functionality and purpose AND the results are published (at least in forms of tables)<br>i. When there is a **small percentage (≤ 10%)** of other products in the same arm, then include<br>ii. When there is a **percentage > 10%** of other products in the same arm AND the results are differentiated by included products, then include<br>iii. When there are different technologies with different functionality and purpose, but which are parts of the same procedure, then include<br>b) Include if the study compares two variants of the same product of the same manufacturer and **change LoE to IV** (case series) – e.g.,<br>i. Comparison of two stents with different size within the same product and **same manufacturer** – 20mm vs. 40mm stent<br>c) Include if the study compares two **different** products within one technology (**keep initial LoE**) – e.g.,<br>i. Two products (stent, valve, etc.) of **different manufacturers**<br>ii. Two products (stent, valves etc.) of the **same manufacturer**	d) The intervention within the PICO of the publication is not of interest<br>i. Imagining studies (with the comparison of two or more imagining modalities)<br>e) In the methods, there is a notice on the permission to use more than one product AND the percentage of cases with the mix is not stated<br><br>There is only one arm with the mix of technologies with similar functionality and purpose > 10 % **AND the results are NOT differentiated by included technologies**
**C**	*Not restricted*	-
**O**	*Not restricted - Efficacy, effectiveness, safety, healthcare related quality of life, surrogate, other: learning effects*	*costs if no other endpoints are considered*
**T**	***from*** *the year with the first hospital case minus 2 years, until to date*	***until*** *the year with the first hospital case minus 2 years*
**S**	*Primary and secondary studies: interventional and observational studies (e.g., studies based on registry data), reviews, systematic reviews, meta-analyses, guidelines, HTAs*	*Cost-effectiveness models, protocols, conference abstracts, editorials, Letter to the Editor, commentary, opinions, errata, notes, technical descriptions*
**Other**	*Language: German, English*<br>*Full text available*<br>*Results of the study are published*	*Language: OTHER THAN German, English*<br>*Full text NOT available*<br>*Results NOT published*

[1] The table was compiled by the authors
[2] Technology specific inclusion criteria are available upon request.

Figure 3.1 Classification of evidence - excerpt of proceeding rules by G-BA 2. chapter § 11 (3) [4, 5]

§ 11 Klassifizierung und Bewertung von Unterlagen

(1) Die Auswertung der Unterlagen besteht aus einer Evidenzklassifizierung nach den Absätzen 2 bis 4 und einer Qualitätsbewertung nach den Absätzen 5 bis 7.

(2) Bei der Klassifizierung der Unterlagen zu diagnostischen Methoden gelten folgende Evidenzstufen:

I a	Systematische Übersichtsarbeiten von Studien der Evidenzstufe I b
I b	Randomisierte kontrollierte Studien
I c	Andere Interventionsstudien
II a	Systematische Übersichtsarbeiten von Studien zur diagnostischen Testgenauigkeit der Evidenzstufe II b
II b	Querschnitts- und Kohortenstudien, aus denen sich alle diagnostischen Kenngrößen zur Testgenauigkeit (Sensitivität und Spezifität, Wahrscheinlichkeitsverhältnisse, positiver und negativer prädiktiver Wert) berechnen lassen
III	Andere Studien, aus denen sich die diagnostischen Kenngrößen zur Testgenauigkeit (Sensitivität und Spezifität, Wahrscheinlichkeitsverhältnisse) berechnen lassen
IV	Assoziationsbeobachtungen, pathophysiologische Überlegungen, deskriptive Darstellungen, Einzelfallberichte, u. ä.; nicht mit Studien belegte Meinungen anerkannter Expertinnen und Experten, Berichte von Expertenkomitees und Konsensuskonferenzen

(3) Bei der Klassifizierung der Unterlagen zu therapeutischen Methoden gelten folgende Evidenzstufen:

I a	Systematische Übersichtsarbeiten von Studien der Evidenzstufe I b
I b	Randomisierte klinische Studien
II a	Systematische Übersichtsarbeiten von Studien der Evidenzstufe II b
II b	Prospektive vergleichende Kohortenstudien
III	Retrospektive vergleichende Studien
IV	Fallserien und andere nicht vergleichende Studien
V	Assoziationsbeobachtungen, pathophysiologische Überlegungen, deskriptive Darstellungen, Einzelfallberichte, u. ä.; nicht mit Studien belegte Meinungen anerkannter Expertinnen und Experten, Berichte von Expertenkomitees und Konsensuskonferenzen

39

Verfahrensordnung Stand: 6. Juli 2018

Appendix 4: Screening of records obtained from registries

The results of the searches via the WHO interface and in the Clinicaltrials.gov database were extracted into Excel spreadsheets. They were then screened based on the PICO aspects using the inclusion and exclusion criteria described above and compared with studies already identified and included via the database search with regard to relevance and availability of publications. The following algorithm was applied in the screening for eligibility of the identified records:

Screening algorithm for eligibility

a) Screening of the (1) intervention AND (2) condition AND (3) title AND (4) registry entry of the trial for eligibility

   i. If not relevant, document in a separate column at the beginning of the registry entry the number of the reason AND

   ii. In a separate sheet the reason dedicated to the number documented in the initial table

   iii. Mark the font of the letters of the excluded entry dark grey

b) **Screening of the registry entry with available publications for relevance**

   i. screening of the publication with the protocol for eligibility of the study; if such publication is not available, one of publications with results

Filter of title AND study design for identification of RCTs

c) Filter the title AND intervention of the entry for „random" or „RCT"

   i. Mark identified entries yellow (the whole row)

   ii. **if publications available:** Download all publications and safe in a folder with the number of the registry entry AND mark the NUMBER of the entry with available publications green

   iii. **In no publications available:** mark the NUMBER of the entry without available publications red

Identified records of RCTs with the start date before 2017 with and without a publication were compared with publications on RCTs identified through database searches. RCTs with a registry entry but without any publication were also documented. RCTs or registry studies from 2017 on were counted and the number documented - RCTs and registry studies were documented separately.

Appendix 5: Data extraction

Data on included studies was extracted per publication of LoE 1-4 studies and technology using a standardized extraction sheet in Excel. Relevant endpoints were defined before the extraction. During the extraction, it was noted if results of relevant endpoint were reported. The actual results were not extracted. Tables 5.1 to 5.3 provide information on extracted variables per technology and type of research (individual study – table 5.1, systematic review/meta-analysis/HTA – table 5.2, clinical guideline – table 5.3). The following algorithms were applied in the decision on the type of LoE, study population, and follow-up:

a) Level of evidence (LoE) [4]
   a. if a study contains two arms and one of the arms includes a potentially concurrent technology, in this case the study is a comparative trial (e.g., stent + DEB vs DEB
   b. if a study contains two arms but the comparator is a variation of the same product (20mm Stent vs 40mm Stent) than the study is a non-comparative LoE IV study.
b) Study population at the beginning of the study (Intervention vs. Control group): total number of study participants; number of study participants per group [intervention vs. control group]
c) Median Follow-up (if available, otherwise follow-up of the publication; in months) – number of study participants at follow-up
d) Follow-up-rate (at time point x: number & percent; IF AVAILABLE: Intention to Treat (ITT): number & per cent; Per-Protocol-Analysis (PPA): number & per cent)

The evaluation of results is based on conclusions from the abstract and from the text

e) If no conclusion in text is available, summary of results from the discussion section was extracted

The extraction of recommendations from guidelines was conducted using a simplified extraction sheet in Word. The extraction was performed by one researcher. Any uncertainties were discussed and consented in the working group.

Table 5.1 Extracted information per publication (individual study)

**Extracted information per publication (individual study)**
**Reference & date**
Author (year)<br>Year of first (online) publication<br>For comparative studies: date of final publication in journal
**Information on topic & study design**
Study acronym, if available<br>Products/technologies (manufacturer) in the intervention arm<br>Products/technologies (manufacturer) in the comparison arm, if available<br>Level of evidence (LoE)
**Study population & Follow-up**
Number of patients at baseline (separately for intervention and comparison arms, if available)<br>Average duration of observation/follow-up in publication in months (with a note, if planned follow-up in study was longer)<br>Number of patients at the end of the observation period (if relevant)<br>Follow-up rate (if relevant)
**Notice if endpoints are reported (pre-determined per technology)**
Mortality<br>Adverse events (safety)<br>Morbidity (method-specific)<br>Health related quality of life<br>Surrogate endpoints<br>Other endpoints
**Conclusions**
Conclusion of the authors from the abstract<br>Conclusion of the authors from the main text (or from the discussion if no conclusion was available as a section)
**Source of funding for the study**

Source: Compiled by the authors

Table 5.2 Extracted information per publication (systematic review, meta-analysis, HTA)

**Extracted information per publication (systematic review - SR, meta-analysis - MA, HTA)**
**Reference & date**
Author (year)<br>Date of publication
**Information on topic & study design**
Products/technologies (manufacturer) in the intervention arm AND Products/technologies (manufacturer) in the comparison arm, if available<br>Type of work (SR, MA, HTA)<br>Number of included trials by level of evidence (LoE)<br>Listing of included randomized controlled trials (RCT, by acronym or reference)<br>Level of evidence (LoE) of the work (1a or 2a)
**Study population & Follow-up**
Minimum follow-up<br>Maximum follow-up<br>Number of patients included in all trials, per RCT, per technology
**Notice if endpoints are reported (pre-determined per technology)**
Mortality<br>Adverse events (safety)<br>Morbidity (method-specific)<br>Health related quality of life<br>Surrogate endpoints<br>Other endpoints
**Conclusions**
Conclusion of the authors from the abstract<br>Conclusion of the authors from the main text (or from the discussion if no conclusion was available as a section)

Source: Compiled by the authors

Table 5.3 Extracted information per guideline

**Extracted information per guideline**
**Reference & Background**
Author (year)<br>Guideline institution<br>Country of the guideline<br>Target group of the guideline
**Targeted technology**
Technology (products/manufacturers) from our sample identified in the guideline<br>Indication
**Recommendation**
Level of Evidence (LoE)/Grade of Recommendation (GoR)/ Relationship between evidence and recommendation of the guideline<br>Recommendation

Source: Compiled by the authors

Appendix 6: Risk of bias (RoB) assessment of RCTs

Risk of bias (RoB) assessment of identified randomized controlled trials (RCTs) was performed at study level based on the criteria of the FJC (p. 164ff, [4]) and methods of the Institute for Quality and Efficiency in Health Care (IQWiG) (p. 170ff [6]) and documented in a standardized Word table as presented in table 6.1. The FJC/IQWiG RoB criteria are in line with the Cochrane Collaboration RoB 1 criteria for RoB assessment at study level. The evaluation was performed by one person. In cases where the assessment criteria „adequate generation of the randomization sequence“ or „concealment of group allocation“ could be answered neither with „yes“ nor with „unclear“, the evidence level was downgraded from 1b to 2b. The quality of systematic reviews and HTAs was not assessed.

Table 6.1 Risk of bias (RoB) tool used for RoB assessment

**Trial**			**Blinding**				
	**Adequate generation of the randomization sequence**	**Concealment of the group allocation**	**Patient/Person**	**Attending or referral physician/personnel**	**Outcome-independent reporting of all relevant endpoints**	**Absence of other aspects**	**Potential risk of bias at study level**
Trial X	yes/no/ unsure	yes/no/ unsure	yes/no/ unsure	yes/no/ unsure	yes/no/ unsure	yes/no	high/low

Source: Based on [4–6]

Appendix 7: Description of the regression model

Description of the variable with weighted cumulative evidence – the "new variable"

The new variable X_{tj}, „results of available body of evidence" at year t, with $t \in T$ representing the technology specific observation period $T_j = \{= \min\,(t(start_j) - 2); \dots; 2017\}$, which starts two years prior to the first procedure documentation of technology j, in year ($t(start_j) - 2$), but at the earliest in 2003, and ends at the latest in 2017. j represents the specific technology $j \in J$ with $J = \{NUBs\ (see\ appendix\ 1, pp. 4 - 7)\}$. The new variable incorporates all identified comparative analyses (LoE 1-3) of a technology published up to and including year t, weighted by category of study results $r \in R = \{positive, negative, neutral\}$ and LoE. The new variable X is decribed by the equation (1).

$$X_{tj} = \sum_{i=(t(start_j)-2)}^{t} w_{rl} x_{ijrl} \qquad (1)$$

The variable was calculated based on LoE 1-3 studies, following the assumption that controlled designs can rather provide reasonably reliable insights on the effects of a technology and are therefore more likely to influence utilization. For example, a systematic review of RCTs (LoE 1a) with negative results was assigned a weight of „-6", and a systematic review (LoE 1a) with positive results was assigned a weight of „+6". A publication reporting no difference between the comparator technologies (categorized as „neutral") received a weight of 1 independently of LoE. Inconclusive results and results of LoE 4 studies were not incorporated into the model. The weight *w* assigned to each category of study results and Level of Evidence (*r, l*) combination are as follows:

- Systematic review of RCTs (LoE 1a) with negative results: -6
- Systematic review of RCTs (LoE 1a) with positive results: +6
- Randomized controlled trial (LoE 1b) with negative results: -5
- Randomized controlled trial (LoE 1b) with positive results: +5
- Systematic review of non- randomized controlled trials (LoE 2a) with negative results: -4
- Systematic review of non- randomized controlled trials (LoE 2a) with positive results: +4
- Non- randomized controlled trial (LoE 2b) with negative results: -3
- Non- randomized controlled trial (LoE 2b) with positive results: +3
- Retrospective controlled study (LoE 3) with negative results: -2
- Retrospective controlled study (LoE 3) with positive results: +2
- Publication reporting no difference between comparator technologies (neutral, LoE 1-3): 1

Three technologies – MVAC, DCB-AV and FD-ULV (for more information on the respective technologies, see appendix 1) – were not considered in the regression analysis since no publications of comparative studies were identified.

Derivation of the regression model

$$Y_{tj} = \beta_{0j} + \beta_{1j}X_{1tj} + e_{tj} \quad (2)$$

$$\beta_{0j} = \gamma_{00} + \gamma_{01}Z_j + u_{0j} \quad (3)$$

$$\beta_{1j} = \gamma_{10} + \gamma_{11}Z_j + u_{1j} \quad (4)$$

$$Y_{tj} = \gamma_{00} + \gamma_{01}Z_j + \gamma_{11}X_{1tj}Z_j + \gamma_{10}X_{1tj} + u_{1j}X_{1tj} + u_{0j} + e_{tj} \quad (5)$$

$$\begin{aligned} number\ of\ procedures_{tj} &= \gamma_{00} + \gamma_{01}technology_j \\ &+ \gamma_{11}results\ of\ available\ body\ of\ evidence_{tj}technology_j \\ &+ \gamma_{10}results\ of\ available\ body\ of\ evidence_{tj} \\ &+ u_{1j}results\ of\ available\ body\ of\ evidence_{tj} + u_{0j} + e_{tj} \quad (6) \end{aligned}$$

As shown in equation 2, Y_{tj} denotes the predicted outcome variable *number of hospital procedures* of technology $j \in J = \{NUBs\ (see\ appendix\ 1, pp. 4-7)\}$. in the year $t \in T_j = \{2003 = \min\left(t(start_j) - 2\right); \ldots; 2017\}$; β_{0j} denotes the technology-individual intercept (random intercept, equation 3); and β_{1tj} represents the random slope, which can vary across both technologies (j) and time points (t). This allows for the possibility of different slopes for each technology over time. β_{1j} also denotes the regression coefficient for the explanatory variable $X_{ij} = results\ of\ available\ body\ of\ evidence$ for technology j in the year t (equation 4) that vary by the grouping variable $Z = technology$. Equations 3 and 4 are substitutes for β_{0j} and β_{1j} in equation 2 resulting in equation 5 and 6. The u_{0j} and u_{1j} are random residual error terms. The residual error term of the fixed effect e_{tj} is normally distributed around zero with variance σ_e^2 $[e_{tj} \sim N(0, \sigma_e^2)]$ [7]. Residual terms u_j are independent from the error term e_{tj}. Model estimation was performed with RStudio (Version 1.4.1717) and lme4 package (analytic code is available upon request). The aim of the regression function is to estimate whether the development of utilization follows the direction of study results, but not to explain the whole variance; this would not be possible by including only one explanatory variable, and many of potential influencing factors [8] are difficult to quantify.

Appendix 8: Results of the search for evidence per database and screening step

Table 8.1 Results of the search for evidence (individual studies, systematic review, meta-analysis, HTA)

Technology	MEDLINE (Ovid)		EMBASE (Ovid)		PubMed		Cochrane Library		Total hits per technology			Included after screening			
	Date	Records	Date	Records	Date	Records	Date	Records	Before duplicate removal	After duplicate removal	HTA	Title/ Abstract	Full text	HTA	Included for analysis
MT	18.05.2019	2971	18.05.2019	6719	24.05.2019	903	20.05.2019	701	11294	7970	48	1226	189	5	194
DCB-AV	18.05.2019	2781	18.05.2019	6128	24.05.2019	598	20.05.2019	793	10300	6548	241	16	2	0	2
DCB-CV												593	132	4	136
DCB-IV												14	4	0	4
DCB-LLV/ULV												419	87	3	87
TAVI	20.05.2019	2034	20.05.2019	3898	24.05.2019	606	22.05.2019	894	7432	5991	179	1243	492	6	498
PECLA/iLA	24.05.2019	1786	24.05.2019	3736	24.05.2019	388	21.05.2019	618	6528	4668	62	215	34	1	35
FD-ULV	20.05.2019	1002	20.05.2019	1570	24.05.2019	170	22.05.2019	310	3052	4014	324	64	7	0	7
DEB-TACE	18.05.2019	1281	18.05.2019	2977	24.05.2019	275	20.05.2019	761	5294	3770	55	1058	151	0	151
EABO	19.05.2019	1107	19.05.2019	2371	24.05.2019	461	22.05.2019	412	4351	3702	248	109	16	0	16
BVS	18.05.2019	1434	18.05.2019	2881	25.05.2019	280	21.05.2019	758	5353	3403	75	1007	226	2	228
EL-P/ICD	17.05.2019	1264	17.05.2019	2440	24.05.2019	486	20.05.2019	288	4478	3308	57	432	61	0	61
MVAC	03.10.2019	1520	03.10.2019	2883	03.10.2019	1671	03.10.2019	125	6199	3191	110	80	10	2	10
F-TUR	18.05.2019	1732	18.05.2019	2864	24.05.2019	256	20.05.2019	183	5035	3178	24	407	44	1	45
LVRC	19.05.2019	1255	19.05.2019	2339	25.05.2019	58	19.05.2019	329	3981	2978	9	196	18	2	20
DES-LLV	18.05.2019	584	18.05.2019	1625	24.05.2019	192	22.05.2019	270	2671	2372	666	567	43	4	47
DES- ULV													53	2	55
VAD	17.05.2019	782	11.06.2019	1917	24.05.2019	235	20.05.2019	251	3185	2238	24	644	76	2	78
SE-BMS	18.05.2019	528	18.05.2019	1705	24.05.2019	73	21.05.2019	103	2409	1916	145	165	15	0	15
ER-ABL	20.05.2019	356	20.05.2019	1214	24.05.2019	159	22.05.2019	409	2138	1768	98	167	13	0	13
ACD	18.05.2019	548	18.05.2019	986	24.05.2019	77	20.05.2019	203	1814	1150	16	336	86	0	86
ACT	18.05.2019	277	18.05.2019	789	25.05.2019	51	20.05.2019	116	1233	894	9	166	29	1	30
IABC	22.05.2019	348	22.05.2019	635	25.05.2019	65	21.05.2019	169	1217	779	16	239	27	0	27
HCO	22.05.2019	1043	22.05.2019	1768	24.05.2019	12	22.05.2019	392	3215	2611	25	187	15	0	15

Table 8.2 Results of the search for guidelines, HTA, safety notifications and in clinical trial registers

Abbreviation of technologies (N=25)	Safety notifications (total)	At least one recall (total)	Safety notifications in Germany (DE)	At least one recall (DE)	Guidelines	HTA	RCT (register)
ACD	0		0		1	0	0
ACT	0		0		1	1	0
BVS	48	yes	2	no	1	2	10
DCB-AV	0		0		2	0	0
DCB-CV	0		0		1	4	>1
DCB-IV	0		0		1	0	0
DCB-LLV	2	no	0		2 (DCB-ULV/LLV)	2 (DCB-ULV/LLV = 3)	2
DCB-ULV	26	yes	7	no	2 (DCB-ULV/LLV)	2 (DCB-ULV/LLV = 3)	>3
DEB-TACE	3	yes	0		1	0	45
DES-LLV	20 (coronary stents)	yes	3 (coronary stents)	no	1 (DES-LLV/ULV)	4 (DES-LLV/ ULV =5)	5
DES-ULV	13	yes	3	yes	1 (DES-LLV/ULV)	4 (DES-LLV/ ULV =5)	19
EABO	38	yes	5	yes	0	0	0
EL-P/ICD	6	yes	1	yes	0	0	0
ER-ABL	0		0		2	0	0
FD-ULV	0		0		0	0	0
F-TUR	2	yes	1	no	13	1	6
HCO	3	yes	1	yes	2	0	1
IABC	0		0		0	0	1
LVRC	0		0		0	2	2
MT	10	yes	2	no	2	5	2
MVAC	0		0		0	2	2
PECLA/iLA	1	yes	1	yes	2	1	1
pVAD	4	yes	3	yes	2	2	9
SE-BMS	0		0		1	0	3
TAVI	74	yes	12	yes	5	12	>1
total					40	40	

Source: Compiled by the authors

Appendix 9: Characteristics of included studies

The largest studies were identified for BVS and TAVI with 141.324 and 24.020 participants, respectively. At least one study with more than 1000 participants was identified for 12 out of the 25 technologies; these were most commonly retrospective registry studies (8/12 technologies), but also RCTs (BVS, TAVI), prospective non-randomized controlled trial (EABO) and case series (DCB-CV).

The longest follow-up was identified in studies of four technologies: with 118 months (ACT), followed by 108 months (DEB-TACE), 106 months (EABO) and 100 months (F-TUR). ACD is the only technology for which only studies focusing on periprocedural outcomes and short follow-up were identified.

Mortality, morbidity, and adverse events were assessed in many studies for all technologies except ACD, for which studies included only surrogate endpoints (and adverse events). Health related quality of life (HRQoL) was assessed in studies on 11 technologies, primarily on cardiovascular indications.

Figure 9.0.1 Randomized controlled trials and results of Risk of Bias (RoB) assessment

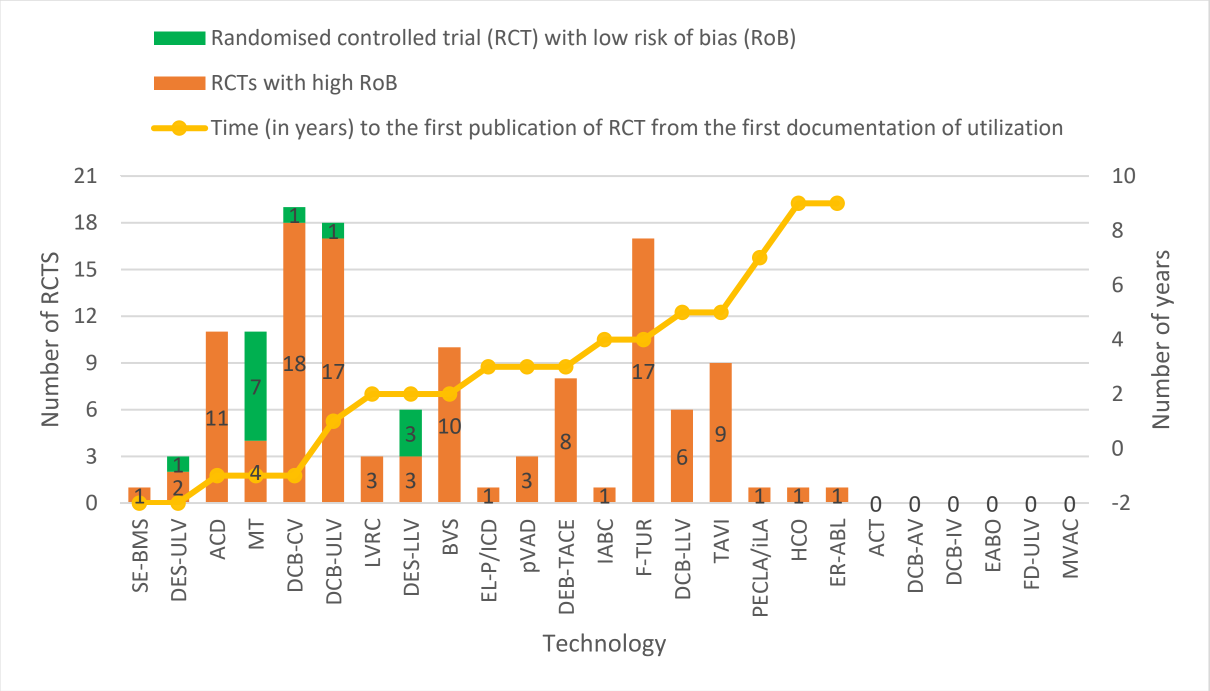

Source: Created by the authors

Table 9.1 Number of included publications with systematic reviews and randomized controlled trials

Abbreviation of technologies (N=25)	Systematic reviews	RCT (Number of publications)
ACD	3	11
ACT	3	0
BVS	26	23
DCB-AV	0	0
DCB-CV	10	33
DCB-IV	25	0
DCB-LLV	10	7
DCB-ULV	17	22
DEB-TACE	2	9
DES-LLV	13	9
DES-ULV	10	4
EABO	1	0
EL-P/ICD	0	1
ER-ABL	0	1
FD-ULV	0	0
F-TUR	10	20
HCO	0	1
IABC	5	2
LVRC	3	4
MT	24	13
MVAC	1	0
PECLA/iLA	8	1
pVAD	3	10
SE-BMS	0	3
TAVI	25	39
total	199	213

Source: Compiled by the authors

Appendix 10: Development of body of evidence

Figure 10.1 Evidence development: Share of publications of LoE 1-3 studies on all publications and its trend of grow by anatomical system and technology

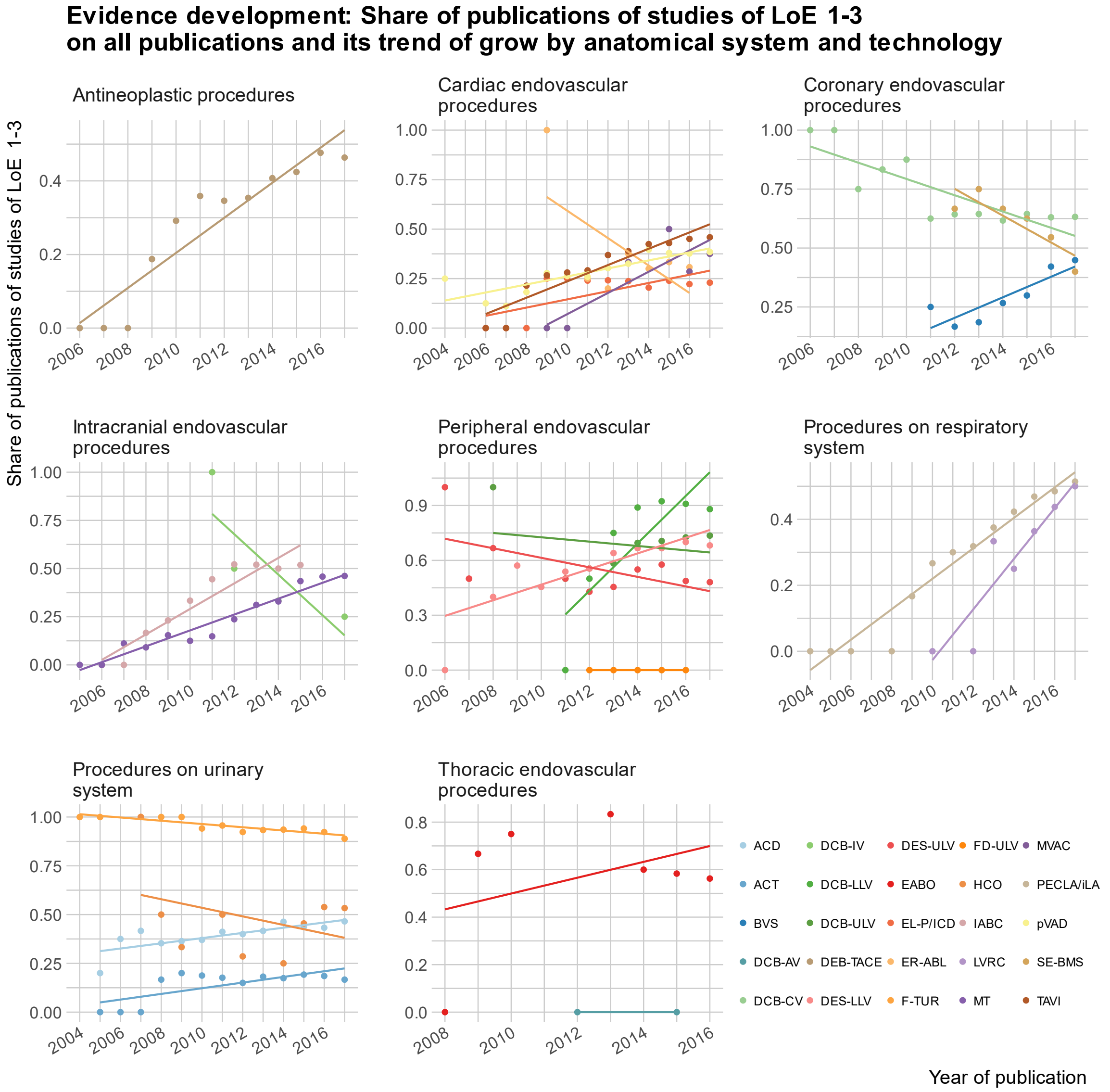

Source: Created by the authors

Figure 10.2 Evidence development: Increase of accumulated number of publications LoE 1-3 and LoE 4 studies by anatomic system and technology

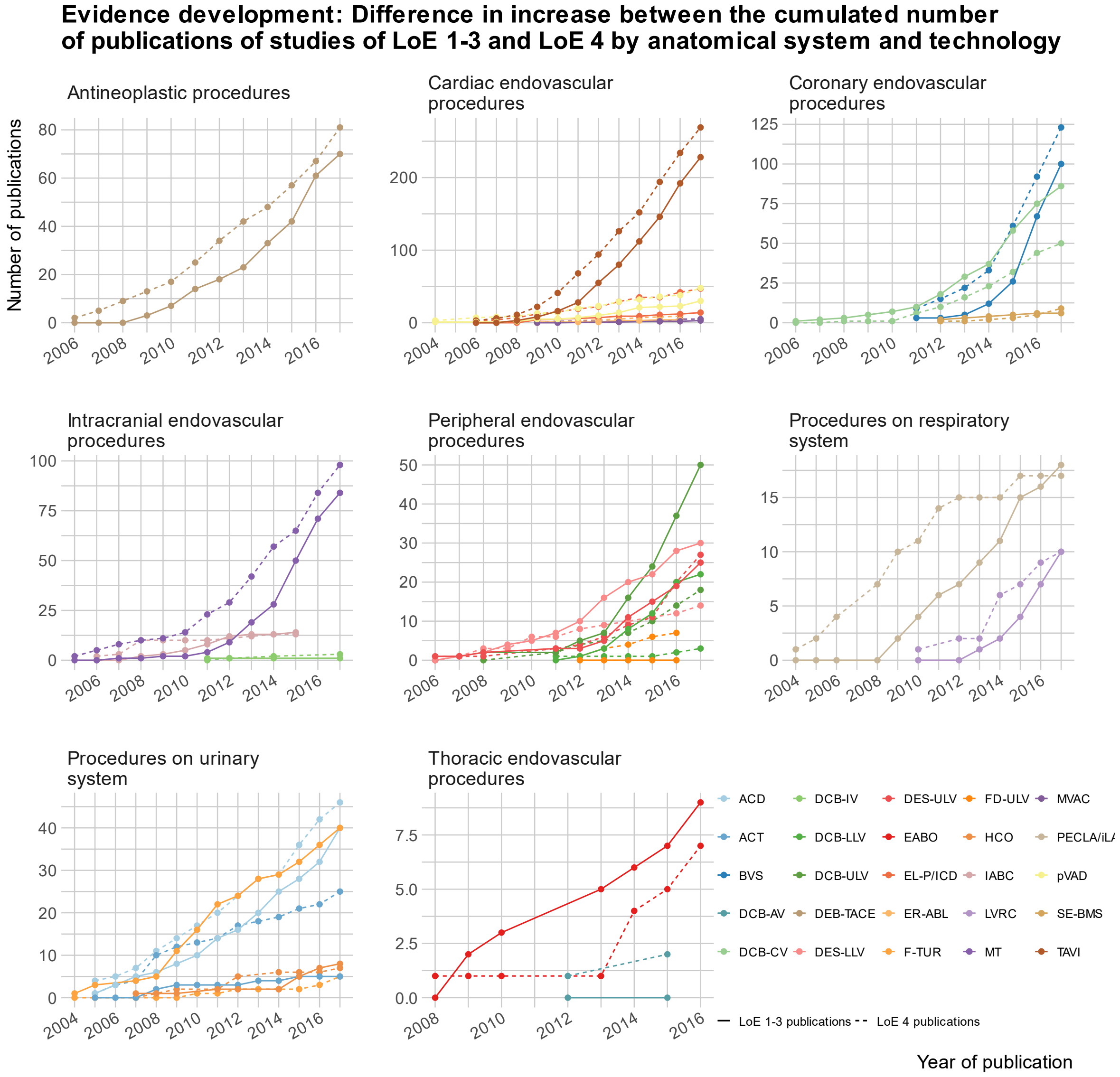

Source: Created by the authors

Appendix 11: Results of evidence for technologies in sample

Figure 11.1 Negative or inconclusive results in publications by LoE and year of publication based on all technologies of sample

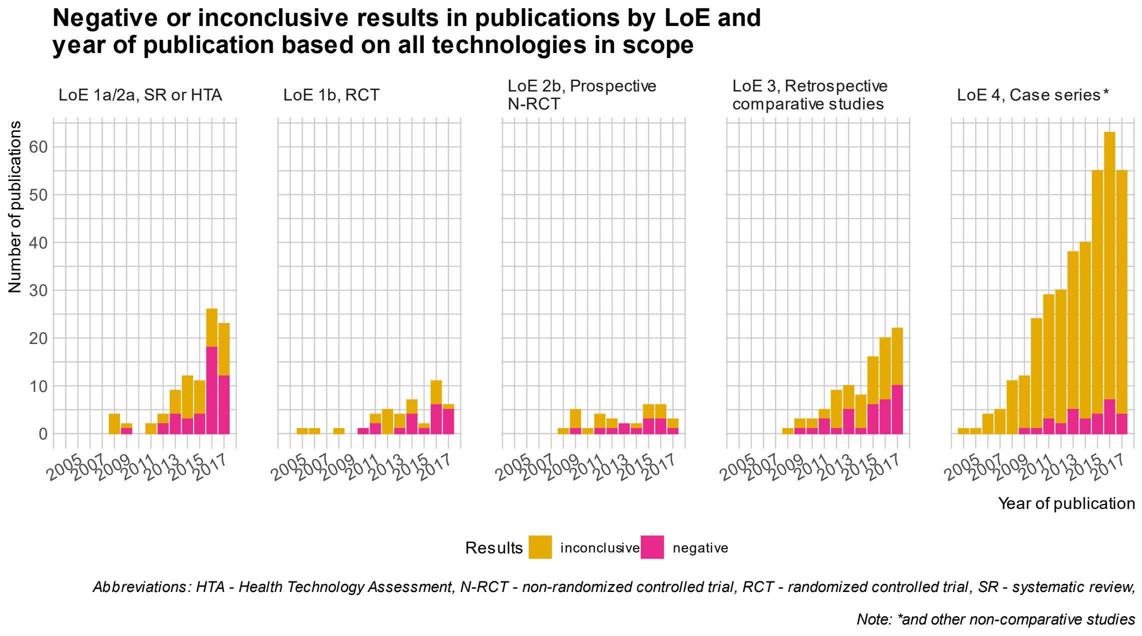

Source: Created by the authors

Figure 11.2 All categories of results in publications by LoE and year of publication based on all technologies of sample

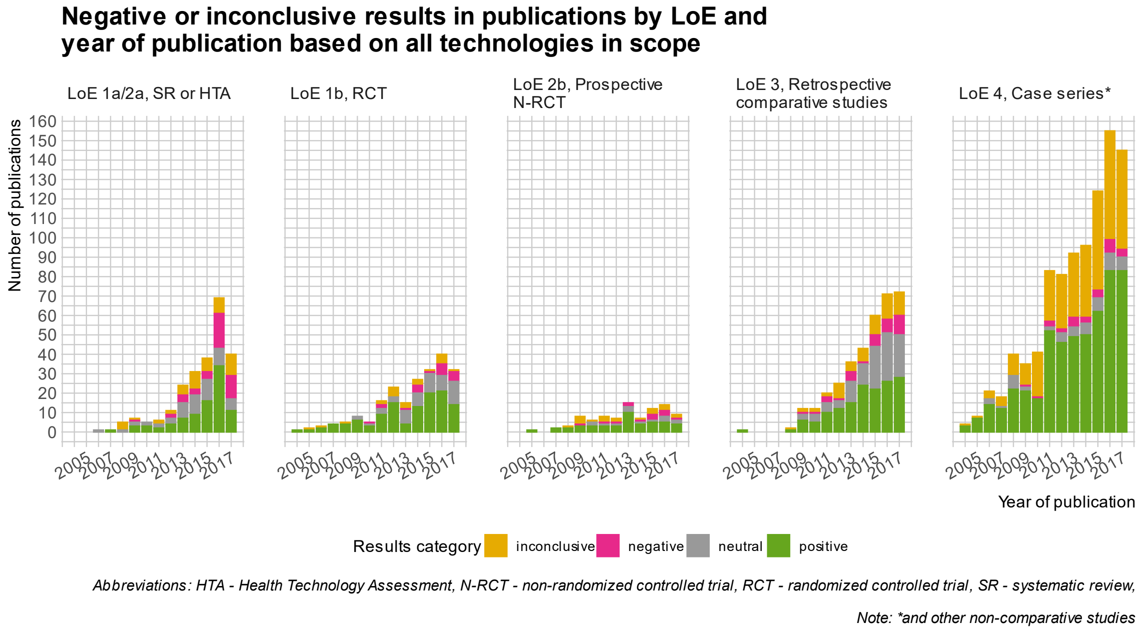

Source: Created by the authors

Figure 11.3 (A-C) Results in publications presented by LoE, year of publication and technology (by availability of negative results)
Source: Figures 11.3 (A-C) were created by the authors

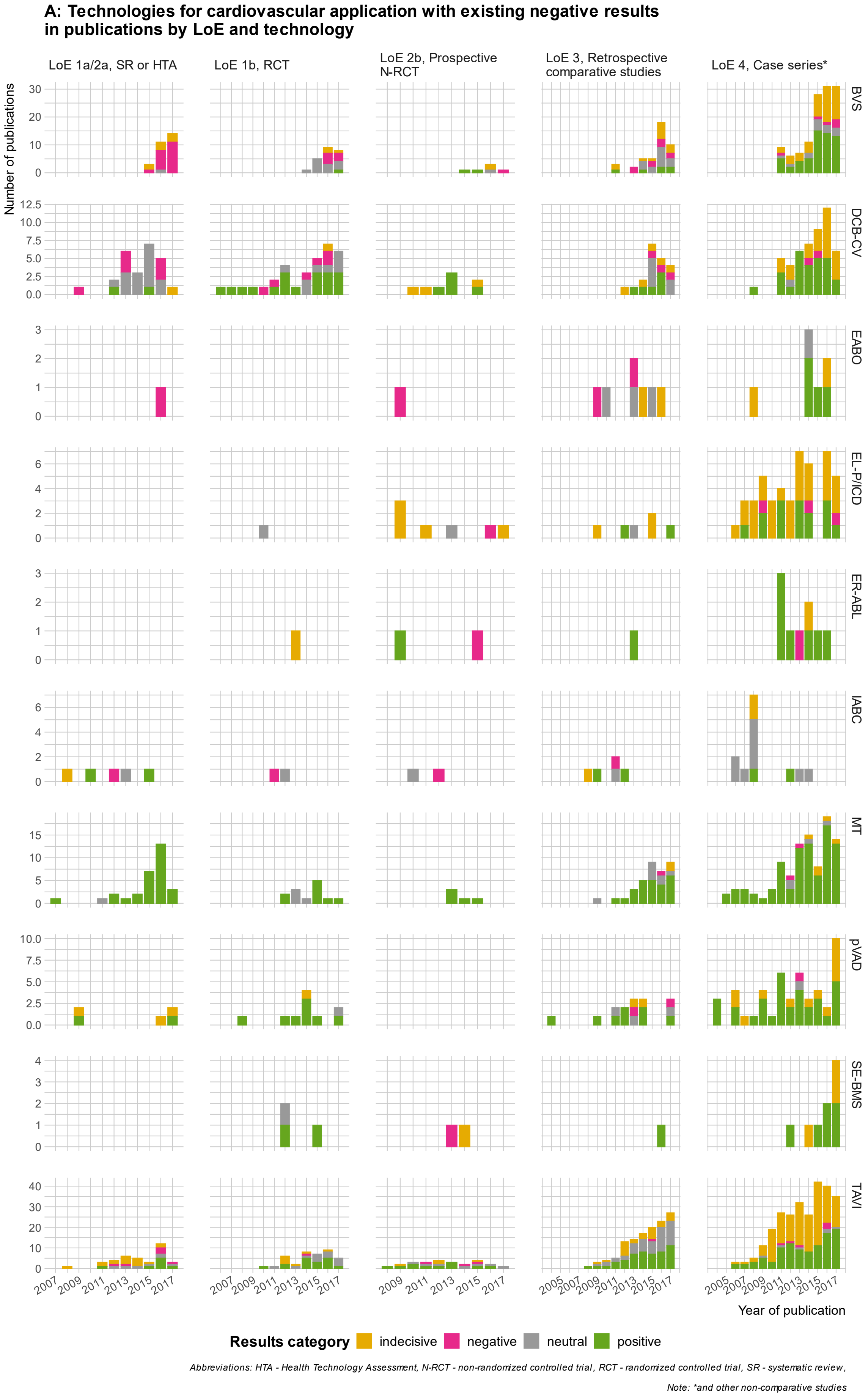
A: Technologies for cardiovascular application with existing negative results in publications by LoE and technology
LoE 1a/2a, SR or HTA
LoE 1b, RCT
LoE 2b, Prospective N-RCT
LoE 3, Retrospective comparative studies
LoE 4, Case series*
Number of publications
BVS
DCB-CV
EABO
EL-P/ICD
ER-ABL
IABC
MT
pVAD
SE-BMS
TAVI
Year of publication
Results category
indecisive
negative
neutral
positive
Abbreviations: HTA - Health Technology Assessment, N-RCT - non-randomized controlled trial, RCT - randomized controlled trial, SR - systematic review,
Note: *and other non-comparative studies

B: Technologies for application in peripheral vessels, urinary tract and other extracorporal application with existing negative results in publications by LoE and technology

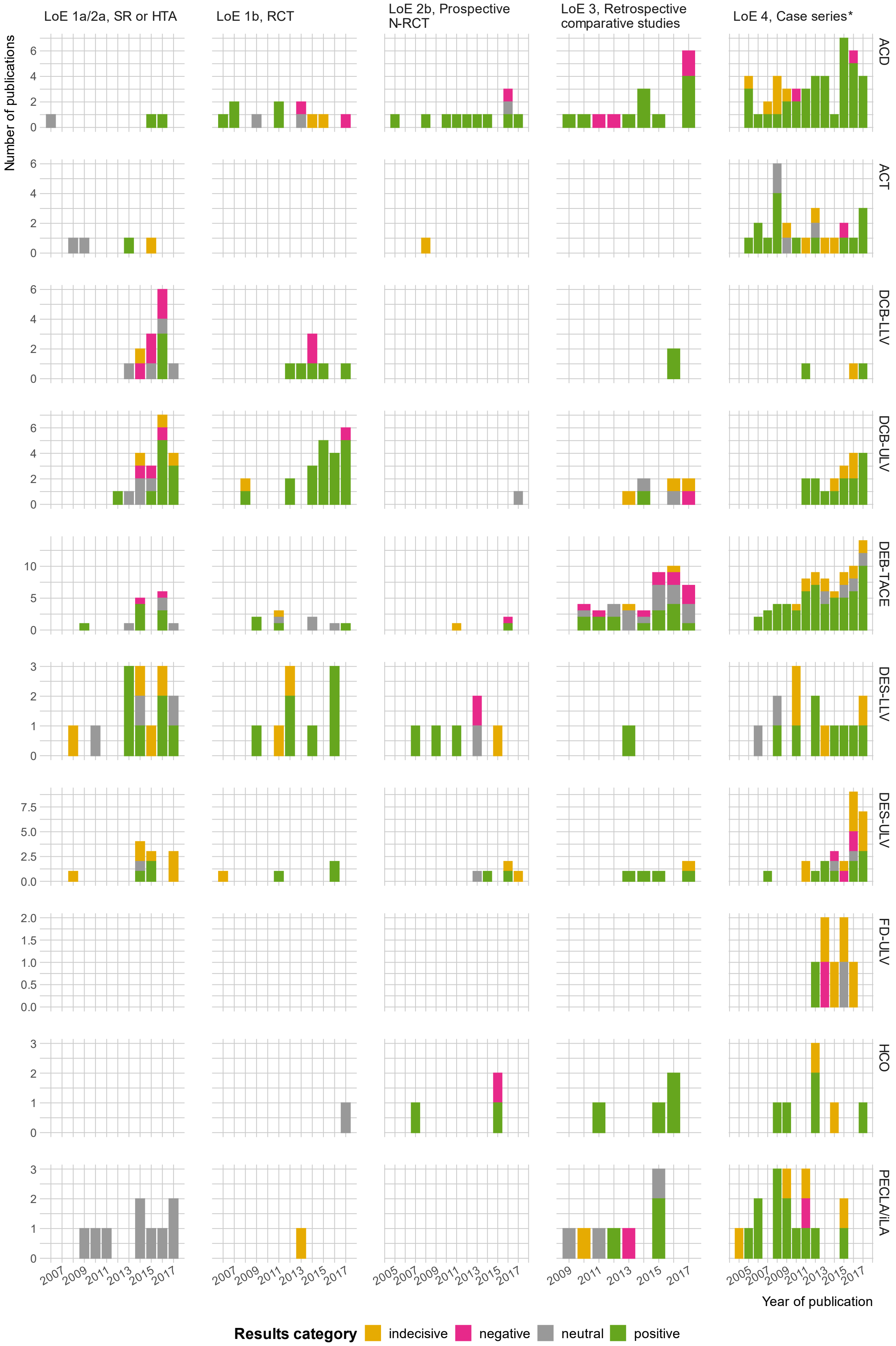

Abbreviations: HTA - Health Technology Assessment, N-RCT - non-randomized controlled trial, RCT - randomized controlled trial, SR - systematic review,

*Note: *and other non-comparative studies*

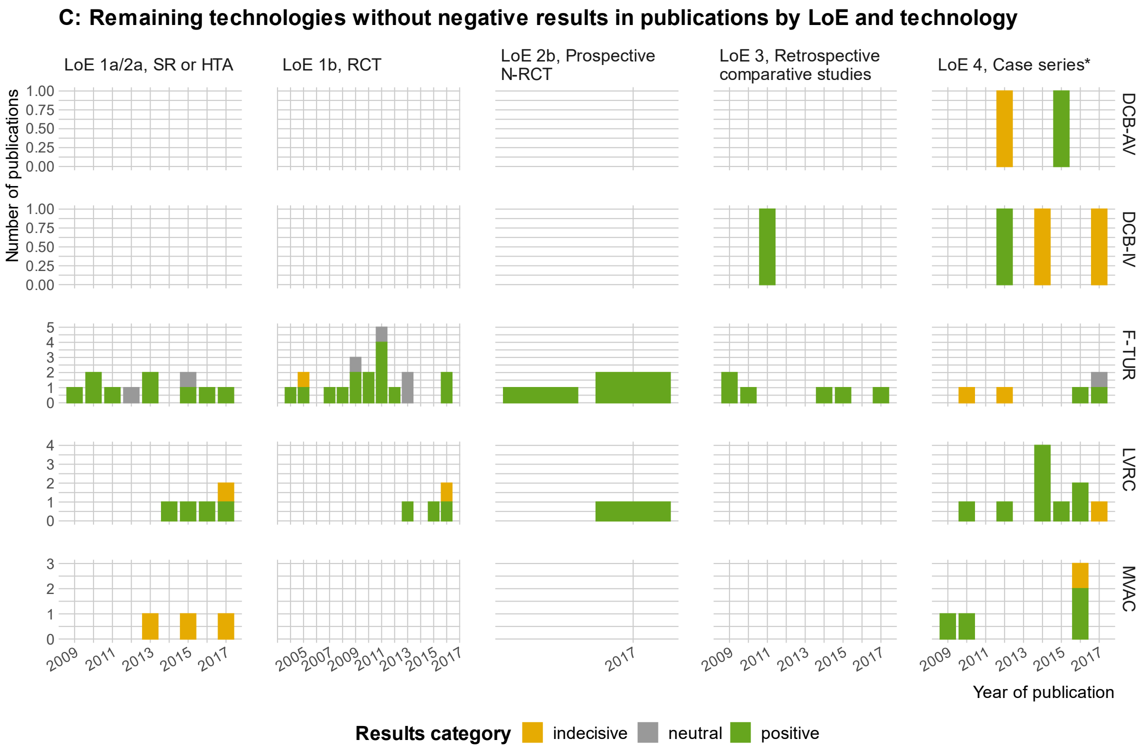

Abbreviations: HTA - Health Technology Assessment, N-RCT - non-randomized controlled trial, RCT - randomized controlled trial, SR - systematic review,

*Note: *and other non-comparative studies*

Appendix 12: Recommendations from clinical guidelines

Positive recommendations were identified for 11 out of 19 technologies with available clinical guidelines, but are predominantly so-called „can“ recommendations, in which the use of a technology is supported under certain conditions (figure 12.1). For eight out of 19 technologies, guideline authors did either not recommend the technology for general use or found the technology inferior to standard care. For all but one technology with more than one available guideline update, these recommendations did not change considerably over time. For pVAD, the recommendation changed from „no recommendation for general use” in 2010 [9] to a „can” recommendation in 2017 [10]. Guideline recommendations do not contradict the prevailing body of evidence at the time of recommendation for any of the 19 technologies.

Figure 12.1 Recommendations from identified clinical guidelines

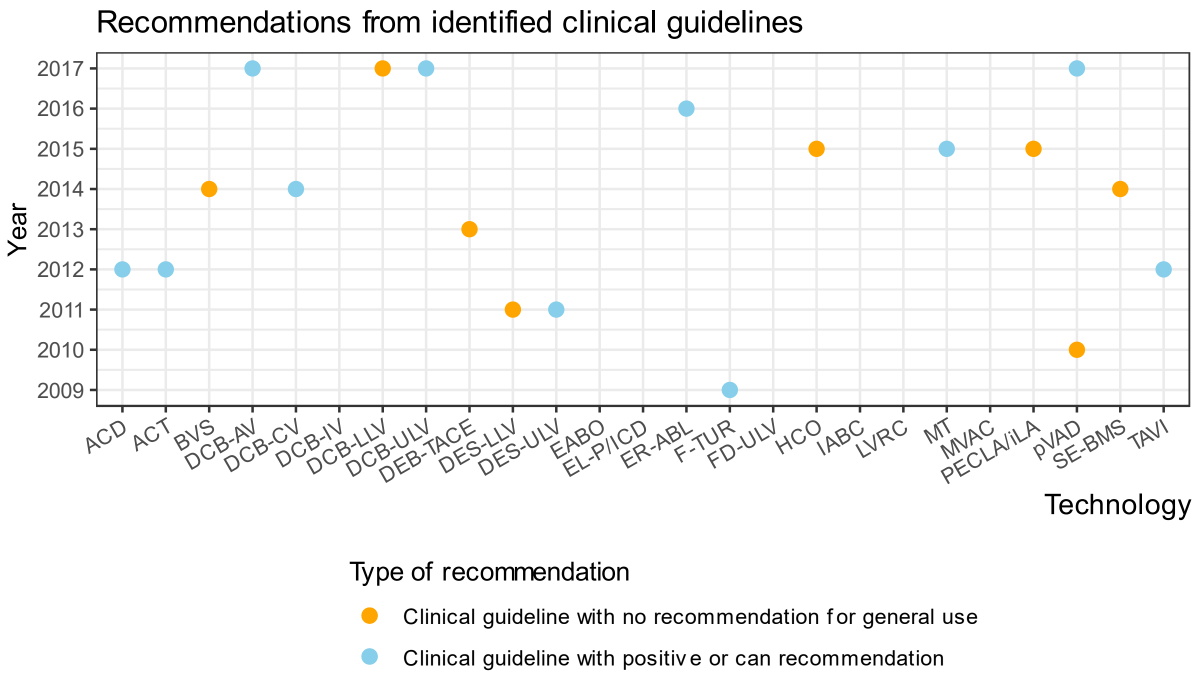

Source: Created by the authors

Appendix 13: References

References

1. Dreger M, Eckhardt H, Felgner S et al. (2021) Implementation of innovative medical technologies in German inpatient care: patterns of utilization and evidence development. Implement Sci 16:94. https://doi.org/10.1186/s13012-021-01159-3
2. Research Data Centre (RDC) of the Federal Statistical Office and Statistical Offices of the Federal States (2018) Hospital statistics based on diagnosis-related groups (DRG statistics), survey years 2005-2017, controlled remote data processing. RDC of the Federal Statistical Office and the statistical offices of the Länder (RDC)
3. Bundesinstitut für Arzneimittel und Medizinprodukte (2023) Code-Search in OPS online. https://www.bfarm.de/EN/Code-systems/Classifications/OPS-ICHI/OPS/Code-search/_node.html. Accessed 16 Jun 2023
4. Gemeinsamer Bundesausschuss (2019) Verfahrensordnung (VerfO) des Gemeinsamen Bundesausschusses (G-BA) in der Version vom 06.03.2019: in der Fassung vom 18. Dezember 2008 veröffentlicht im Bundesanzeiger Nr. 84a (Beilage) vom 10. Juni 2009 in Kraft getreten am 1. April 2009 zuletzt geändert am 16. August 2018 veröffentlicht im Bundesanzeiger BAnz AT 05.03.2019 B2 in Kraft getreten am 6. März 2019
5. Gemeinsamer Bundesausschuss (2018) Verfahrensordnung (VerfO) des Gemeinsamen Bundesausschusses (G-BA) in der Version vom 05.07.2018: in der Fassung vom 18. Dezember 2008 veröffentlicht im Bundesanzeiger Nr. 84a (Beilage) vom 10. Juni 2009 in Kraft getreten am 1. April 2009 zuletzt geändert am 16. März 2018 veröffentlicht im Bundesanzeiger BAnz AT 04.07.2018 B1 in Kraft getreten am 5. Juli 2018
6. Institut für Qualität und Wirtschaftlichkeit im Gesundheitswesen (2017) Allgemeine Methoden: Version 5.0, Version 5.0. Institut für Qualität und Wirtschaftlichkeit im Gesundheitswesen (IQWiG), Köln
7. Hox JJ (2010) Multilevel analysis: Techniques and applications, 2. ed. Quantitative methodology series. Routledge Taylor & Francis, New York
8. Balas EA, Chapman WW (2018) Road Map For Diffusion Of Innovation In Health Care. Health Affairs 37:198–204. https://doi.org/10.1377/hlthaff.2017.1155
9. Deutschen Gesellschaft für Kardiologie - Herz- und Kreislaufforschung (2010) Infarkt-bedingter kardiogener Schock: Diagnose, Monitoring und Therapie. Leitlinie der Deutschen Gesellschaft für Kardiologie - Herz- und Kreislaufforschung
10. Ibanez B, James S, Agewall S et al. (2017) 2017 ESC Guidelines for the management of acute myocardial infarction in patients presenting with ST-segment elevation: The Task Force for the management of acute myocardial infarction in patients presenting with ST-segment elevation of the European Society of Cardiology (ESC). Eur Heart J 39:119–177. https://doi.org/10.1093/eurheartj/ehx393
